# Selection of the optimal long-acting injectable formulation of ivermectin for use in humans to target malaria vectors in Western Africa: evaluation of pharmacokinetics and mosquitocidal efficacy in cattle under laboratory conditions

**DOI:** 10.1186/s13071-026-07263-x

**Published:** 2026-05-06

**Authors:** Lamidi Zela, Sié Hermann Pooda, Angélique Porciani, Samuel Beneteau, André Barembaye Sagna, Sophie Le Lamer-Déchamps, Nicolas Moiroux, Cheick Oumar Wendpagnandé Ouédraogo, Anyirékun Fabrice Somé, Cédric Pennetier, Christophe Roberge, Adrien Marie Gaston Belem, Koumbobr Roch Dabiré, Guiguigbaza-Kossigan Dayo, Karine Mouline

**Affiliations:** 1 Centre International de Recherche-Développement sur l’Elevage en zone Subhumide (CIRDES), Bobo-Dioulasso, Burkina Faso; 2 Université Nazi Boni, Bobo-Dioulasso, Burkina Faso; 3 Université Daniel-Ouezzin Coulibaly, Dédougou, Burkina Faso; 4 Infectious Diseases and Vectors: Ecology, Genetics, Evolution and Control (MIVEGEC), University of Montpellier, IRD, CNRS, INRAE, Montpellier, France; 5 Independant Statistician, Montpellier, France; 6 Institut de recherche pour le developpement (IRD), Antenne de Bobo-Dioulasso, Bobo-Dioulasso, Burkina Faso; 7 MedinCell, Montpellier, France; 8 Pôle de Zoologie Médicale, Institut Pasteur de Dakar, Dakar, Sénégal; 9 Institut de Recherche en Sciences de la Santé (IRSS), Direction Régionale de l’Ouest, Centre National de Recherche Scientifique et Technologique, Bobo-Dioulasso, Burkina Faso; 10 Université Cheikh Anta Diop, Dakar, Sénégal; 11 Present Address: Centre de Recherche et de Veille sur les Maladies Vectorielles dans la Caraïbe (CRVC), ASTRE, Université of Montpellier, CIRAD, INRAE, Petit Bourg, Guadeloupe, France

**Keywords:** Malaria vector control, Residual transmission, *Anopheles* mosquitoes, Insecticide resistance, Ivermectin, Long-acting injectable ivermectin, Pharmacokinetics, Mosquitocidal efficacy

## Abstract

**Background:**

Ivermectin, a semisynthetic endectocide, is widely used against parasitic nematodes in humans and animals. Its lethality to *Anopheles* mosquitoes that have fed on treated hosts represents a promising malaria control strategy, particularly against outdoor transmission. However, standard oral formulations for use in humans produce short-lived mosquitocidal blood concentrations, limiting epidemiological impact. To meet WHO Preferred Product Characteristics (PPC) for endectocides against malaria vectors (hazard ratio [HR] > 4 for at least 1 month), we developed three long-acting injectable ivermectin formulations (LAIFs) based on BEPO® depot technology (MedinCell, Jacou, France) and compared these in cattle to identify the most suitable candidate for future use in humans.

**Methods:**

A cattle–*Anopheles* model was used under laboratory conditions in Bobo-Dioulasso, Burkina Faso. Three LAIF candidates (mdc-STM-001, mdc-STM-002, mdc-STM-003) were injected into calves (*n* = 5 per formulation) at 0.6 mg/kg body weight, with untreated calves as controls (*n* = 5). Plasma ivermectin concentrations were measured over 130 days post-injection and analyzed using non-compartmental pharmacokinetics. Direct skin feeding assays were conducted at 15 time points (days 2–126 post-injection) using insecticide-susceptible *Anopheles gambiae *Kisumu (KIS) and wild-derived resistant (VK5) *Anopheles* colonies. Efficacy was assessed based on 10-day cumulative mortalities, HRs, 50% lethal concentrations (LC50) and duration of exposure above the 10-day LC50, also accounting for the extrinsic incubation period of *Plasmodium falciparum*.

**Results:**

All formulations were well tolerated. The mdc-STM-001 formulation showed the most favorable pharmacokinetic (PK) profile, with a controlled peak concentration (Cmax) of 34.5 ± 12.7 ng/ml and the lowest inter-individual variability (12%). Ten-day HRs exceeded 4 and cumulative mortalities were > 50% for at least 60 days in both strains. Median mosquito lifespan remained below 10 days for at least 90 days post-injection. The 10-day LC50 for resistant mosquitoes (3.66, 95% confidence interval 2.69–4.97 ng/ml) was maintained for ≥ 126 days.

**Conclusions:**

The mdc-STM-001 formulation was identified as the optimal candidate for future use in humans. A single injection induced sustained mosquitocidal efficacy for at least 2 months, achieving HR > 4 against both susceptible and resistant *Anopheles* populations and meeting WHO PPC for malaria endectocides. Although extrapolation from cattle to humans requires caution, the favorable PK profile and robust entomological outcomes support progression to phase 1 clinical trials. Ivermectin’s established safety record further strengthens the rationale for clinical development.

**Supplementary Information:**

The online version contains supplementary material available at 10.1186/s13071-026-07263-x.

## Background

Malaria remains a leading global health concern with an estimated 282 million clinical cases and 610,000 deaths in 2024, over 95% of which occurred in Sub-Saharan Africa [1]. Significant gains in the fight against the disease have been made between 2000 and 2015 [2], mainly due to indoor residual spraying of houses and universal coverage with pyrethroid insecticide-treated nets, two core vector control interventions recommended by the WHO [3]. However, progress has stalled in the last 10 years [4], notably coinciding with widespread, growing resistance in *Anopheles* vector populations, which is considered to be due to the huge selection pressure exerted by continuous insecticide-based interventions and massive pesticide use in agricultural practices [5]. Resistance refers to both (i) physiological resistance, involving reduced susceptibility to the insecticidal molecule through target-site mutations or metabolic detoxification processes [6] and (ii) behavioral (qualitative) resistance, as defined by Carrasco et al. [7], whereby mosquitoes avoid exposure to insecticides spatially (by biting outdoors), temporally (by biting early in evening or morning when humans are outside their nets or housing) or trophically (by feeding on non-human hosts). New generations of long-lasting impregnated nets (LLINs) incorporating dual active ingredients or synergists [8, 9] have been recently recommended by the WHO to circumvent pyrethroid resistance [10]. However, these tools still primarily target nocturnal, indoor-biting *Anopheles* mosquitoes, and are bypassed by behaviorally resistant vectors [11, 12]. The resurgence in malaria cases in recent years [1] highlights the urgent need for novel and complementary strategies to reinforce existing interventions and reverse this concerning trend. In particular, tools targeting the diverse biting behaviors of *Anopheles* vectors, whether innate or induced by interventions, are essential for addressing this challenge [7, 13].

Mass drug administration (MDA) to humans of the ivermectin endectocide is one approach that has been proposed to overcome limits of current malaria vector control methods [14, 15]. Acting systemically, this molecule targets internal parasites as well as external blood-feeding arthropods, including *Anopheles* malaria mosquitoes. It thus primarily reduces the survival probability of* Anopheles* mosquitoes, considered to be the most critical determinant of their vectorial capacity [16]. Because the insecticide is delivered through the host’s bloodstream, it functions regardless of any spatial or temporal blood-feeding adaptations of mosquitoes. Ivermectin is the leading endectocide candidate for vector control, notably because of its strong, well-established safety profile from nearly 4 decades of extensive clinical use in MDA campaigns against lymphatic filariasis and onchocerciasis [17]. This safety profile reflects its selective action on glutamate-gated chloride channels unique to invertebrates [18], a mechanism of action that is distinct from those of the different insecticide classes used in public health against malaria vectors. Consequently, ivermectin is a potent vector control tool that could mitigate current physiological resistance issues.

All *Anopheles* species and strains tested to date have been shown to be susceptible to ivermectin, although the degree of susceptibility varies among species [19]. Moreover sub-lethal effects are also interesting to transmission control as they negatively impact fecundity and fertility [20, 21], locomotor and biting behaviors [22, 23] or *Plasmodium* parasite development [24, 25]. MDA of ivermectin to humans may therefore effectively reduce residual malaria transmission.

Various oral drug dose regimens have been used during randomized controlled trials (RCTs) that have tested ivermectin MDAs against malaria. Standard oral ivermectin dosing for parasitic disease treatment in humans (150–200 µg/kg body weight) achieves lethal plasma levels for *Anopheles* mosquitoes for fewer than 7 days [26]. In field studies from sub-Saharan Africa, this regimen administered every 3 weeks during the rainy season to populations in southern Burkina Faso resulted in a significant 20% reduction in malaria incidence among children under the age of 5 years [27]. Increased dosing regimens (1 × 400 µg/kg and 3 × 300 µg/kg, i.e. 300 µg/kg daily for three consecutive days) prolong ivermectin persistence in the host bloodstream and its mosquitocidal efficacy up to 14–21 days with good safety [28, 29]. Modeling studies have predicted that 80% coverage of the target population using such a regimen has the potential to reduce malaria incidence by 30% to 70%, depending on the transmission settings [30].

In field studies, the BOHEMIA RCT in Mozambique and Kenya applied the 1 × 400 µg/kg regimen monthly for 3 consecutive months across the rainy season [31]. In Kenya, this led to reduced malaria incidence in children aged 5–15 years by 26% compared to the control arm [32]. However, in Mozambique, the same regimen failed to produce a significant reduction [33]. Two other RCTs testing the 3 × 300 µg/kg dose administered monthly in combination with either dihydroartemisinin–piperaquine (DHAP) in the Galápagos Islands (MATAMAL, treatments for three consecutive months [34]) or seasonal malaria chemoprevention (SMC) in Burkina Faso (RIMDAMAL II, treatments for four consecutive months [35]) also showed no significant epidemiological impact. Possible explanations for such lack of effects include biological factors related to vector populations, such as poor translation of mosquitocidal effects from laboratory studies to the field or overlooked aspects of wild mosquito bionomics in the trial [36]. Pharmacokinetic (PK) data from the RIMDAMAL II trial also revealed a shorter-than-expected residence time of lethal plasma ivermectin, as drug levels dropped below detection in nearly 50% of participants by day 14 [35]. In Mozambique, logistical challenges increased the duration of the administration rounds so that population coverage never reached the threshold expected for efficacy. However, all of these trials did demonstrate the safety of increased and repeated dosing of oral ivermectin.

Both PK limitations and operational challenges encountered in the fields are intrinsic to oral dosing and highlight the need for long-acting ivermectin formulations that can sustain mosquitocidal effects while allowing logistical flexibility. To date, only a single human-targeted long-acting formulation has been developed—a gastric-resident oral device with approximately 14-day efficacy [37].

In this context, using the BEPO^®^ injectable technology [38], we developed three long-acting ivermectin formulations (LAIFs) for human use in malaria endemic regions. These formulations were designed according to the efficacy target set by the WHO recommendations for preferred product characteristics of endectocides in malaria vector control [39], which specify a hazard ratio (HR) > 4 for mosquitoes feeding on treated hosts for at least 1 month post-dose. We then used a cattle–mosquito colony model [21, 40], exposing both insecticide-susceptible and pyrethroid-resistant *Anopheles* strains through direct skin feeding assays, thereby assessing the formulations’ performance in the presence of potential cross-resistance or tolerance mechanisms. Our main objective was to compare PK, pharmacodynamic (PD), and mosquitocidal efficacy outcomes among the three candidate formulations to identify the optimal one for subsequent clinical evaluation in human.

## Methods

### Study site

All experiments were conducted in Bobo-Dioulasso, south-western Burkina Faso, through a collaboration between the Institut de Recherche en Sciences de la Santé (IRSS) and the Centre International de Recherche-Développement sur l’Élevage en zones Subhumides (CIRDES). Both institutes have controlled insectary facilities, and CIRDES also maintains stables for cattle husbandry.

### Mosquito colonies

Two *Anopheles* mosquito colonies were used in this study:

(i) *Anopheles gambiae *Kisumu (KIS). This colony is fully susceptible to all classes of insecticides. It is maintained in the IRSS insectaries, and is routinely monitored for contamination by other species. It is screened for insecticide resistance using molecular techniques [41, 42]. For the experiments, mosquitoes were transported in meshed cages covered with a moist cloth to CIRDES. Transport duration was approximately 10 min.
(ii) *Anopheles coluzzii* VK5 (VK5). This colony was established at CIRDES in November 2020, prior to the start of the experiments in January 2021. A total of 150 blood-fed females were collected from the village of VK5 in the Kou Valley (approx. 50 km northwest of Bobo-Dioulasso), an irrigated rice-growing area where different classes of pesticides are used in agriculture [43]. The use of pyrethroid-impregnated bed nets is high due intense mosquito nuisance. The mosquito’s bio-ecology is well characterized, with *An. coluzzii* representing > 95% of* Anopheles* species and permanently found breeding in the irrigated fields. *Anopheles coluzzii* populations in this area are characterized by a high prevalence of resistance to all insecticides classes [44], justifying the choice of this site in the search for an insecticide-resistant colony. Collected females were allowed to individually oviposit. Species identification was performed post-oviposition using established PCR protocols [41]. Only progeny from confirmed *An. coluzzii* were retained for the experiments. As expected, based on the collection site, all females belonged to the target species.

Both* Anopheles *colonies were housed in insectaries that were maintained under similar environmental conditions and maintenance protocol: temperature of 26° C ± 1° C, relative humidity of 75% ± 5% and photoperiod of 12/12-h light/dark [40]. The VK5 colony’s pyrethroid resistance status was routinely monitored by sampling approximately 40 females per generation and screening for the knockdown resistance West African allele (KDR-W; L1014F) of the voltage-gated sodium channel gene using quantitative PCR (qPCR) [42]. For each direct skin-feeding assay time point, females were randomly drawn from at least five different cages to minimize potential cage effects.

### Long-acting ivermectin formulations

Three ready-to-use LAIFs were evaluated in this study: mdc-STM-001, mdc-STM-002 and mdc-STM-003. These were developed using BEPO^®^ technology [38], a proprietary long-acting injectable technology (Medincell, Jacou, France). Following subcutaneous injection, BEPO^®^ forms an in situ bioresorbable polymeric depot via a solvent-exchange mechanism, leading to precipitation of a polymer matrix that enables sustained drug release. Each formulation tested consisted of three components: (i) a biocompatible solvent to ensure injectability; (ii) ivermectin, as the active pharmaceutical ingredient; and (iii) a mixture of two biocompatible and biodegradable polymers that control drug release kinetics. These polymers undergo progressive hydrolysis into bioabsorbable by-products over time due to a simple hydrolysis mechanism. The three LAIFs differ from each other in terms of polymer composition and proportions, and mdc-STM-003 differs from the other two in terms of ivermectin loading.

All formulations were imported into Burkina Faso with clearance from the Direction Générale des Services Vétérinaires of Burkina Faso (Permit No 2020/199) and were stored at room temperature, protected from light, until administration to cattle. The study protocol was approved by the Institutional Animal Ethics Committee of CIRDES (document reference number: 004–10/2020 CEB-CIRDES).

### Cattle hosts and treatments

Twenty-five male calves (Métis strain: Fulani zebus × Baoulé crossbreed) with a median weight of 117 kg (interquartile range [IQR] 106–129 kg) were purchased from villages in the Valley du Kou area 2 months prior to the study to allow for acclimation. Upon arrival at the CIRDES animal facility, the calves were treated for trypanosomiasis and gastrointestinal parasites using Berenil 2000® (MSD Animal Health, Rahway, NY, USA) and Benzal® (LAPROVET S.A.S., France), respectively. Animals were housed outdoors in completely screened enclosures to prevent exposure to or reinfestation with ectoparasites and trypamosomoses. They were fed rice straw and 1 kg/day of cottonseed cake, with water and sea salt provided ad libitum.

Calves were randomly assigned to one of five experimental arms (*n* = 5 calves per arm) using stratified randomization to balance body weight across groups. Four arms received LAIFs, and one served as the untreated control. The formulations were administered via a single subcutaneous injection into the loose skin anterior to the shoulder.

- mdc-STM-001 and mdc-STM-002 LAIFs were each injected as a single dose of 0.6 mg/kg (referred to as mdc-STM-001–0.6 and mdc-STM-002–0.6, respectively).
- mdc-STM-003 LAIF was administered at two different doses: 0.6 and 1.5 mg/kg (referred to as mdc-STM-003–0.6 and mdc-STM-003–1.5, respectively).

The 1.5 mg/kg dose represents the upper limit of feasible subcutaneous volumes for human application and was included to assess dose-dependent effects on pharmacokinetics and mosquitocidal efficacy. Nominal injection volumes per animal ranged from 0.62 to 2.14 ml (detailed in Table 1). The animals' identification numbers; their weight upon arrival at CIRDES and on the day of injection; administered formulations; and injected volumes are provided in Additional file 1: Table S1. Animal health was monitored each day throughout the study period by assessing clinical signs, local injection site reactions, body weight and weight gain.

**Table 1:** Nominal treatment volumes per formulation and dose to be administered to cattle

Number of cattle	LAIF	Ivermectin dose (mg/kg)	Nominal volume (µL/kg)
5	mdc-STM-001	0.6	7.72
5	mdc-STM-002	0.6	7.80
5	mdc-STM-003	0.6	5.85
5	mdc-STM-003	1.5	14.6
5	N/A (control)	0	0

*N/A* not applicable

### Mosquito blood-feeding bioassays

To ensure equivalent ivermectin exposure, mosquitoes from both the KIS and VK5 colonies were simultaneously exposed to the same cattle hosts during blood-feeding. This design enabled direct comparison of susceptibility across colonies under identical conditions. Direct skin blood-feeding assays were conducted following previously established protocols [40]. Prior to feeding, mosquitoes were deprived of sugar for 12 h and provided access to water-soaked cotton only. At each time point, approximately 100–130 females per colony were introduced into net-covered bowls, and the bowl were then held against the shaved flank of the calf for 20 min using a rubber band. Separate bowls were used for each colony. Fully engorged mosquitoes were subsequently distributed into cups (10 females per cup), with a maximum of 120 cups per colony per time point. All cups were maintained under controlled conditions in the CIRDES insectary. Mosquito mortality was recorded daily between 10:00 a.m. and 11:00 a.m. for 30 days post-blood meal. To mitigate positional bias, cups were randomly repositioned on shelves after each observation.

A total of 16 blood-feeding assays were conducted: a pre-treatment (baseline) assay and assays at 2, 7, 17, 21, 28, 42, 56, 70, 84, 91, 98, 105, 112, 119 and 126 days after injection (DAI). Due to limited mosquito availability, KIS mosquitoes were not tested at 7 and 91 DAI. Surplus blood-fed mosquitoes were stored at − 20 °C for downstream analyses.

### Ivermectin bioanalysis and pharmacokinetics

Jugular venous blood samples were collected from treated calves at each mosquito exposure time point and at additional time points (30 samples per animal) to characterize ivermectin pharmacokinetics. The sampling time points included: at pre-dose; at 2, 5 and 12 h post-injection; followed by daily sampling until DAI 4, sampling every 2–4 days until DAI 21 and finally by weekly sampling until DAI 132–133. Blood samples were also collected from control animals at pre-dose and at DAI 1, 14, 28, 56, 84 and 111. Blood samples were centrifuged at + 4 °C (2000 rpm, 10 min) to isolate plasma, which was stored at − 80 °C until shipment for bioanalytical analysis. Ivermectin plasma concentrations were quantified using a liquid–liquid extraction with qualified liquid chromatography–tandem mass spectrometry (LC–MS/MS) methods. Two calibration ranges were used (0.2–20 ng/ml [low range], 5–500 ng/ml [high range]), using K_2_-EDTA as anticoagulant and ivermectin-D_2_ as internal standard. The quality controls (0.6, 10, 16 ng/ml [low range]; 15, 250, 400 ng/ml [high range]) were within the acceptance criteria (precision ≤ 15.00%, deviation ± 15.00%). Incurred sample reanalysis validated ivermectin stability for at least 680 days at − 20 °C. Plasma samples collected from cattle were stored at − 80 °C for no longer than 162 days before analysis and were used to validate the ivermectin concentrations measured for PK analysis.

At study completion, control animals were returned to stock, while treated animals were euthanized. Injection sites and depot with surrounding tissues were sampled to quantify the residual ivermectin and copolymers. These analyses were performed using an ultra-performance liquid chromatography system (limit of quantification 0.9 µg/ml) for ivermectin and nuclear magnetic resonance analysis for copolymers. Both methods were developed for exploratory purposes only.

### Outcomes and analysis

Unless specified otherwise, analysis were performed using R version 4.4.3 (2025–02-28) [45]. Models fit were assessed using the “performance” [46] and DHARMa [47] packages. All estimates are reported with 95% confidence intervals (95% CI) and/or* p*-values.

#### Cattle weight gain

Differences in weight gain between treatment groups throughout the study were analyzed using the Kruskal–Wallis rank sum test, followed by pairwise two-sided exact Wilcoxon rank sum tests with 95% confidence intervals. *P*-values were adjusted using the Holm method ([48]).

#### Pharmacokinetics

Pharmacokinetic parameters of ivermectin were calculated for each LAIF using a non-compartmental analysis (Phoenix® WinNonli® version 8.1; Certara USA, Inc., Princeton, NJ, USA). These included the rate of systemic exposure (Cmax); the time to reach Cmax (Tmax); the extent of systemic exposure over the study duration (area under the curve, AUClast), over 28 DAI (AUC0-28d) and over 91 DAI (AUC0-91d); concentrations at 28 DAI (C28d), 56 days (C56d), 91 DAI (C91d) and at study end (Clast); and the apparent terminal phase half-life (T1/2) when calculable. The systemic exposure parameters were normalized to the actual administered dose, calculated for each calf based on the weight of each syringe before and after injection. The inter-individual variability in plasma concentrations and PK parameters was expressed as the coefficient of variation (CV %) for each experimental group.

#### Mosquito-related outcomes

##### Mosquito blood-feeding rate

For each colony, we compared the mosquitoes’ blood-feeding rates across treatment arms using a generalized linear mixed model (GLMM; [49]) with a binomial error distribution. The outcome variable was the number of blood-fed mosquitoes, with the total number of mosquitoes included as an offset. The model included an interaction term between treatment and DAI, as well as a random intercept for cattle. The model was fitted using restricted maximum likelihood.

##### Times post-feeding

Efficacy outcomes are provided for 4- 10- and 30-days post-mosquito blood-feeding. The 4-day period reflects the acute toxic effect, which typically manifests within 2–3 days post-ivermectin blood meal. The 10-day post-blood-feeding period is used in relation to the 10-day minimum extrinsic incubation period (EIP) needed for the parasite to develop in the mosquito and to reach the salivary glands [50], thereby rendering it infectious and active in transmission. The 30-day follow-up captures the residual effects of ivermectin on surviving mosquitoes, which may include both indirect sublethal effects and potential interactions with the mosquitoes' age. Only data related to the 10-day follow-up period are presented and thoroughly discussed in the main text. Outcomes for the 4-day and 30-day follow-up period are given in Additional file 2: Figures S3, S4.

##### Cumulative mortality

Proportions of dead mosquitoes at 10-days post-blood-feeding were computed for each colony, formulation and DAI.

##### Survival analysis and HRS

Daily mosquito mortality was recorded for up to 30 days post-blood-feeding. For each mosquito colony and DAI, survival data were used to generate Kaplan–Meier survival curves, stratified by treatment group.

Per colony, survival patterns at each time point were compared to that of the control group using Cox proportional hazards models (mixed-effects regression model [51]). The models included “treatment group” and “DAI” as fixed effects, with random intercepts for individual calf ID and mosquito cup (nested random effects) to account for variability within treatment groups. HRs at different time points post-blood-feeding are given to assess the impact of ivermectin on mosquito mortality risks and HR variations over time. Post hoc pairwise comparisons of HRs between time points were adjusted for multiple testing using the Tukey method, implemented via the “emmeans” package [45]

##### Dose–response analysis and coverage duration

Dose–response relationships were analyzed using the “drc” package in R [52], modeling 4-day and 10-day mosquito mortality as a function of host ivermectin plasma concentrations. A four-parameter log-logistic model was fitted with the upper limit fixed at 1. Confidence intervals (CIs) were computed using the tfls method (t from logarithm scale).

## Results

### Cattle follow-up

A mild reaction (agitation) was observed in most animals after the injection, regardless of dose or LAIF administered (data not shown). There were no unscheduled cattle deaths, and no clinical signs were observed in any of the treated groups throughout the study. The major local reaction was swelling, which occurred in all animals, as expected with this LAIF technology. Median weight gain throughout the study (4 months) was unaffected by the test item treatment (Kruskal–Wallis test, *χ*^2^ = 8.4892,* df* = 4, *P*-value = 0.075; Table 2). For each experimental group, monthly weight measures are given in the Additional file 1: Table S2.

**Table 2:** Median cattle weight gain per treatment group with interquartile range (IQR)

	Control	mdc-STM-001-0.6	mdc-STM-002-0.6	mdc-STM-003- 0.6	mdc-STM-003- 1.5
Weight gain (kg) Median (Q1–Q3)	27.4 (25.8–31.6)	37.2 (35.8–44.2)	39.6 (36.6–41.6)	35.3 (31.2–36.2)	30.0 (28.6–40.0)

### Ivermectin pharmacokinetics

**Fig. 1:**
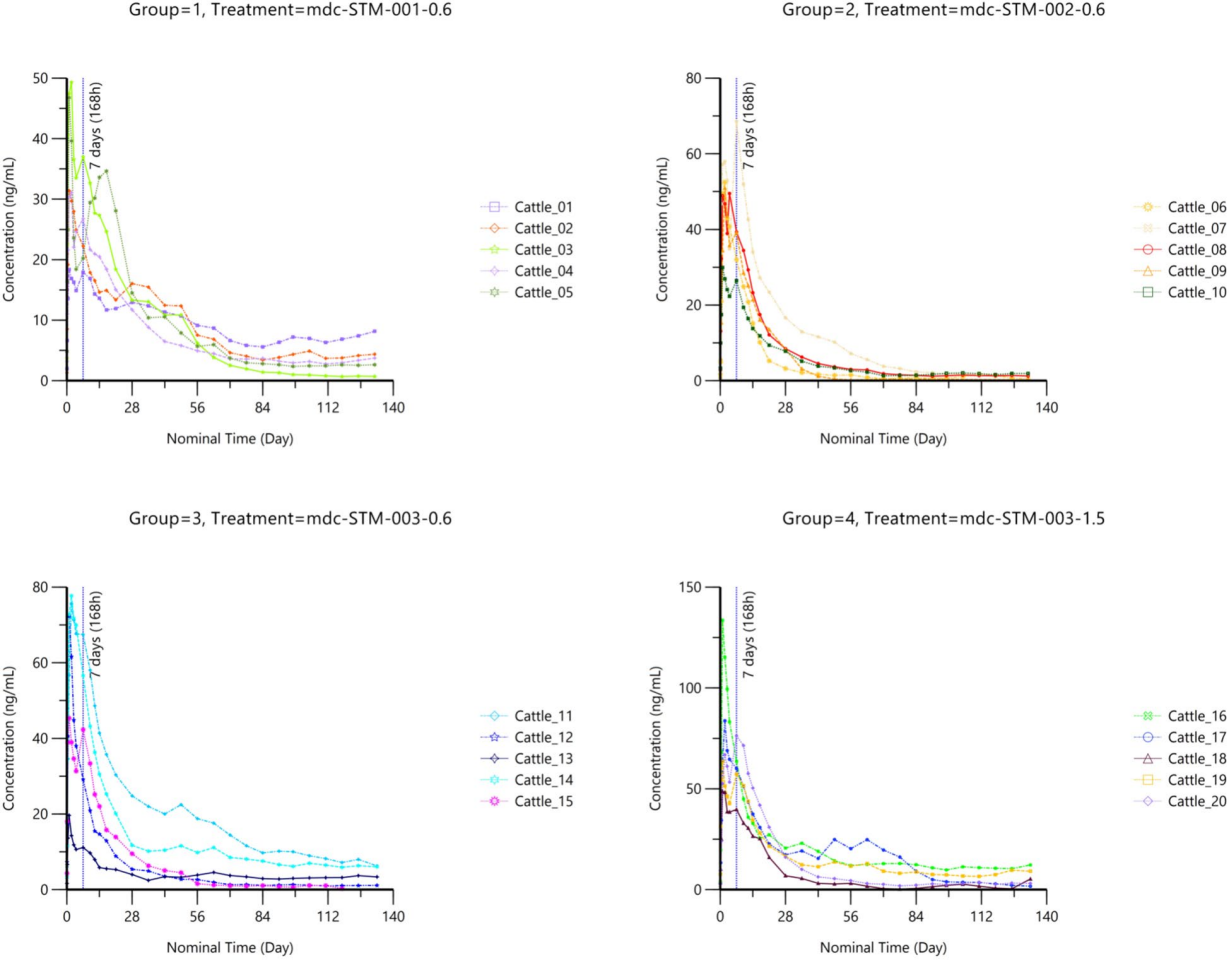
Individual kinetic profiles of ivermectin in cattle plasma after single sub-cutaneous administration of candidate long-acting ivermectin formulations at a dose of 0.6 or 1.5 mg/kg

A total of 635 blood samples were collected, including 600 samples from LAIF-treated cattle (4 treatment groups, 5 cattle per group, *n* = 30 per cattle) and 35 samples from control cattle (1 control group, 5 cattle, *n* = 7 per cattle). Concentration–time patterns were analyzed using data up to DAI = 132 for the mdc-STM-001–0.6 and mdc-STM-002–0.6 formulations, and up to DAI 133 for the mdc-STM-003–0.6 and mdc-STM-003–1.5 formulations. Ivermectin plasma concentrations were analyzed according to the treatment (formulation and dose). Individual concentration–time profiles of ivermectin after LAIF injection are illustrated in Fig. 1. The mean concentration–time profiles over the study duration and for the first 7 days (the latter provided only to better visualize the burst, i.e. peak plasma level) are provided in Additional file 2: Figure S1.

Ivermectin was not detected in any of the samples collected in the control group during the study period nor in any samples collected before ivermectin injection in the treated groups. Following sub-cutaneous injection, ivermectin plasma concentrations were generally measured in all samples collected during the study, with quantifiable levels still present at the final study time point (DAI 132 or 133).

After a single sub-cutaneous administration of LAIFs, the plasma levels of ivermectin increased to reach the maximal level (i.e. the burst; known to occur with long-acting injectables [38]) at 24 h or 48 h post-dose (median Tmax). This was followed by two phases of decrease: a first phase until day 56 (1344 h post-dose), followed by a slower phase characterized by a regular decrease in ivermectin plasma levels until the study end. Among the formulations tested, mdc-STM-001–0.6 showed the lowest rate of decrease during the first phase and the steadiest levels during the second phase. In contrast, the mdc-STM-002–0.6 formulation exhibited the fastest decrease compared to the other LAIFs. The inter-animal variability in ivermectin plasma levels (expressed as coefficient of variation CV%) was moderate. The lowest variability was seen with mdc-STM-001–0.6 (mean CV% 41%, range 12–70%), followed by mdc-STM-003–1.5 (mean 54%, range 22–87). The highest variability was observed for mdc-STM-002–0.6 (mean CV% 62%, range 25–106%) and mdc-STM-003–0.6 (mean 72%, range 34–98%).

The mean ivermectin PK parameters of each LAIF after injection are reported in Table 3. Additional parameters are given in the Additional file 1: Table S3.

**Table 3:** Non compartmental pharmacokinetic parameters of ivermectin in cattle plasma after single sub-cutaneous administration of the different long acting ivermectin formulations (LAIFs) at a dose of 0.6 (mdc-STM-001, mdc-STM-002, mdc-STM-003) or 1.5 mg/kg (mdc-STM-003)

Parameters	mdc-STM-001-0.6	mdc-STM-002-0.6	mdc-STM-003-0.6	mdc-STM-003-1.5
Tmax^a^ (h)	24 (24; 48)	48 (24; 168)	24 (24; 48)	24 (24; 168)
Cmax (ng/mL)	35.40 ± 12.70	50.30 ± 13.70	58.10 ± 25.20	81.20 ± 32.20
Cmax/Dose (ng/mL/mg/kg)	58.30 ± 22.00	84.30 ± 22.60	99.40 ± 42.20	56.10 ± 22.40
Clast (ng/mL)	3.93 ± 2.75	1.03 ± 0.70	3.43 ± 2.74	6.30 ± 4.35
AUC_0–91d_ (h ng/mL)	25055 ± 2936	21121 ± 8866	28890 ± 18668	41820 ± 13286
AUC_0–91d_/Dose (h ng/mL/mg/kg)	40990 ± 5664	35479 ± 14839	49483 ± 31994	28903 ± 9300
C28d (ng/mL)	13.70 ± 1.60	8.90 ± 4.85	11.10 ± 8.30	15.50 ± 5.10
C56d (ng/mL)	6.70 ± 1.67	2.92 ± 2.63	7.34 ± 7.14	10.30 ± 6.90
C91d (ng/mL)	3.48 ± 1.85	1.11 ± 0.84	4.33 ± 3.99	5.55 ± 3.72

For each parameter, values are expressed as mean ± standard deviation (SD). Tmax: time to reach Cmax. Cmax: maximal plasma concentration. Clast: last quantifiable plasma concentration. AUC_0–28d_: area under curve from time 0 to 28 days. AUC_0–28d_/Dose: dose-normalized AUC_0–28d_. AUC_0–91d_: area under curve from time 0 to 91 days. AUC_0–91d_/Dose: dose-normalized AUC_0–91d_. C28d: plasma concentration 28 days post injection. C56d: plasma concentration 56 days post injection. C91d: plasma concentration 91 days post injection

^a^Median (min; max)

At a dose of 0.6 mg/kg, differences in PK parameters emerged across formulations when both the entire study duration and the 91-day period (3 months post-injection, considered to be the best targeted release duration) were considered. These differences involved performance, quantification levels over time and inter-animal variability. The peak plasma concentration (Cmax) for mdc-STM-001–0.6 was at least 30% lower that those of the other LAIFs, with mdc-STM-003–0.6 showing the highest Cmax. This result suggests that mdc-STM-001–0.6 provided a safer burst compared to the other LAIFs. The inter-animal variability in Cmax was moderate for all formulations, with the lowest variability observed for mdc-STM-002–0.6 (CV% 27%) followed by mdc-STM-001–0.6 (CV% 36%) and mdc-STM-003–0.6 (CV% 43%). The overall systemic exposure over the 92 DAI period (AUC_0–91d_) was quite similar for all formulations, with the lowest value observed for mdc-STM-002–0.6 and the highest value observed for mdc-STM-003–0.6. Inter-animal variability in AUC was much lower for mdc-STM-001–0.6 (CV% 12%) than for mdc-STM-002–0.6 (CV% 42%) and mdc-STM-003–0.6 (CV 66%). The mean ivermectin plasma concentrations after each month post sub-cutaneous injection (C28d, C56d, C91d, Clast) decreased slowly until the end of the study for all formulations, remaining close to or above 3.5 ng/ml for mdc-STM-001–0.6 and mdc-STM-003–0.6, and around 1 ng/ml for mdc-STM-002–0.6. There was moderate to high inter-animal variability, ranging from 53% for mdc-STM-001–0.6 to 92% for mdc-STM-003–0.6. Additionally, the ratio of Cmax to the level measured after each month (C28d, C56d, C91d) was up to twofold lower for mdc-STM-001–0.6 compared to other LAIFs at the dose of 0.6 mg/kg.

For mdc-STM-003, both tested doses (0.6 and 1.5 mg/kg) exhibited a hypo-proportional increase in Cmax and AUC that was approximately 40% less than proportionality. However, these findings should be considered cautiously due to the high inter-animal variability observed, particularly at the low dose (Table 3; Additional file 1: Table S2).

Because the second phase of the PK profile was relatively flat, the apparent terminal phase (T1/2) was accurately estimated for only some animals, with a mean T1/2 ranging from 20 days for mdc-STM-003–1.5 to 33 days for mdc-STM-001–0.6. The remaining amounts of ivermectin depot and copolymers were the lowest in animals treated with the mdc-STM-001–0.6 formulation (Additional file 1: Tables S4, S5).

### Mosquitocidal activity evaluation

#### KDR-W status of the VK5 mosquito lots

Generations F2 to F8 of the VK5 colony were used in this experiment. Mosquito batches exposed at 2, 7, 17, 21, 28 and 42 DAI consisted of 74–80% individuals exhibiting pyrethroid-resistant phenotypes (i.e. homozygous or heterozygous for the KDR-W allele of the voltage-gated sodium channel gene [42]). The remaining batches (exposed at 56, 70, 84, 91, 98, 105, 112, 119 and 126 DAI) displayed a lesser proportion of resistant mosquitoes (50–54%). Detailed qPCR results by generation and DAI are given in Additional file 1: Table S6.

#### Blood feeding rate

High blood-feeding rates were consistently observed across all time points for both the KIS and VK5 colonies (Table 4). No statistically significant differences in feeding success were observed between treatment groups or formulations at any time point (data not shown), indicating uniform host attractiveness and feeding opportunity across experimental conditions.

**Table 4:** Blood-feeding rates of KIS and VK5 mosquitoes allowed to feed on cattle from the different treatment groups

	Control	mdc-STM-001-0.6	mdc-STM-002-0.6	mdc-STM-003-0.6	mdc-STM-003-1.5	Total
KIS	0.900 (3908/4341)	0.921 (4128/4481)	0.897 (4206/4487)	0.892 (4080/4572)	0.831 (3675/4421)	0.889 (19817/22302)
VK5	0.966 (5047/5224)	0.945 (5005/5295)	0.935 (5091/5447)	0.961 (5160/5371)	0.968 (5174/5343)	0.955 (25477/26680)

Numbers in brackets indicate the number of fed mosquitoes out of the total exposed

#### Efficacy outcomes

The total number of mosquitoes monitored for survival by colony and treatment group is presented in Table 5. Detailed distribution of both colonies by individual cattle, cup and DAI is provided in Additional file 1: Table S7. Due to limited availability of KIS mosquitoes, no feeding assays were conducted at 7 and 28 DAI. Mosquito husbandry issues affected the same colony at 105 and 119 DAI; these data points were excluded from the analysis. Unexpected high mortality rates during the first 4 days post-blood-feeding (> 0.20) were detected for KIS mosquitoes fed on control calves B328 at 42 DAI (mortality rate 0.437) and B321 at 112 DAI (mortality rate = 0.21); therefore, these mosquito lots were excluded from efficacy analyses.

**Table 5:** Number of KIS and VK5 mosquitoes followed for survival experiments for each treatment group

	KIS	VK5
Control	1926	3161
mdc-STM-001-0.6	2122	3206
mdc-STM-002-0.6	2123	3186
mdc-STM-003-0.6	2135	3207
mdc-STM-003-1.5	2093	3182
Total	10,399	15,942

### Mortality rates

**Fig. 2:**
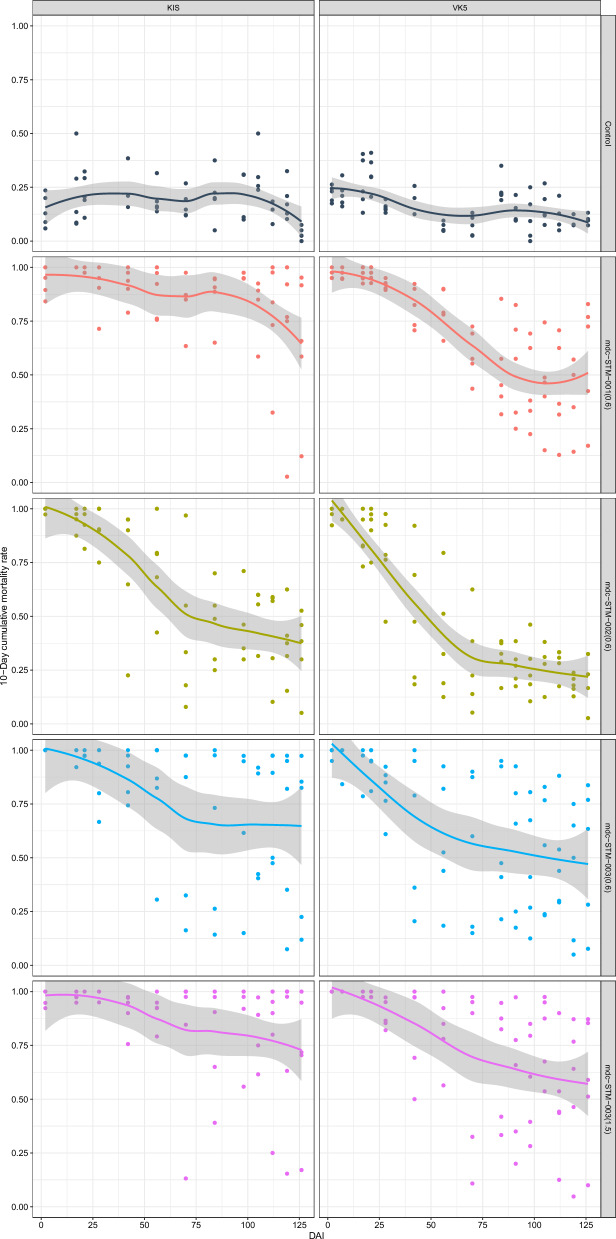
Ten-day cumulative mortality rates of KIS and VK5 mosquitoes fed at different days after injection (DAI) on control or treated cattle. Treated cattle received a single LAIF injection at a dose of 0.6 mg/kg for the mdc-STM-001, mdc-STM-002 and mdc-STM-003 formulations, and at an additional dose of 1.5 mg/kg for the mdc-STM-003 formulation. The smooth line represents mean values estimated by the LOESS (locally estimated scatterplot smoothing) method; the gray area shows the 95% confidence interval [45]. Results for the 4-day cumulative mortalities are shown in Additional file 2: Figure S3. KIS,* Anopheles gambiae* colony Kisumu; LAIF, long-acting injectable ivermectin formulation; VK5,* Anopheles coluzzii* VK5 colony

The mdc-STM-001–0.6 formulation showed the highest efficacy against KIS mosquitoes, comparable to that of mdc-STM-003–1.5 (Fig. 2). From 2 DAI to approximately 100 DAI, over 75% of KIS mosquitoes died within 10 days after feeding on cattle treated with mdc-STM-001–0.6; beyond this period, mortality rates remained > 50%. For VK5 mosquitoes, mdc-STM-001–0.6 maintained ≥ 50% mortality between 2 and 80 DAI, whereas other formulations at the dose of 0.6 mg/kg were less effective. The high dose mdc-STM-003–1.5 formulation achieved the longest duration of efficacy for VK5 mosquitoes, with mortality rates > 50% from 2 to 110 DAI.

Among all formulations, mdc-STM-001–0.6 exhibited the most consistent efficacy profile. Mosquito mortality data for this LAIF showed the lowest inter-animal variability, with fewer deviations from the mean trend across all sampling points.

### Survival and HRs

**Fig. 3:**
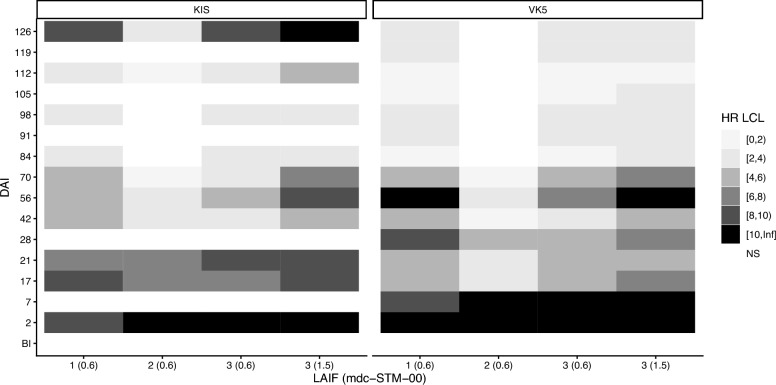
Heat maps illustrating the LCL of 10-day mortality HRs for KIS and VK5 colony mosquitoes fed at different DAI on cattle treated with the formulations mdc-STM-001–0.6, mdc-STM-002–0.6, mdc-STM-003–0.6 or mdc-STM-003–1.5. HRs and *P*-values for each DAI are available in Additional file 1: Table S7. Results for 4- and 30-day HRs are given in Additional file 2: Figure S4. BI, Time point before injection; DAI, days after injection; HR, hazard ratio; KIS,* Anopheles gambiae* colony Kisumu; LAIF, long-acting injectable ivermectin formulation; LCL, lower 95% confidence limit; VK5,* Anopheles coluzzii* VK5 colony

No significant differences in mosquito survival were observed across treatment groups prior to LAIF administration for either the KIS or VK5 colonies (Fig. 3; Additional file 1: Table S8; Additional file 2: Figures S2, S3), confirming no confounding effects from host variability. Following treatment, all formulations administered at the dose of 0.6 mg/kg (mdc-STM-001, mdc-STM-002, mdc-STM-003) and at 1.5 mg/kg (mdc-STM-003) induced a significant mosquitocidal effect. The magnitude and duration of these effects varied according to mosquito strain and formulation but remained statistically significant through the full follow-up period (126 DAI).

The mdc-STM-002–0.6 formulation was clearly the least effective, with HRs ≥ 4 for the shortest duration regardless of colony (Fig. 3). It was therefore excluded from further efficacy comparisons.

The mdc-STM-001–0.6 formulation induced sustained 10-day HR ≥ 4 through 70 DAI for both KIS and VK5 strains. The mdc-STM-003–06 formulation displayed less sustained efficacy, with HRs > 4 at 42 DAI for both the KIS and VK5 colonies. The higher dose of the mdc-STM-003 formulation ( mdc-STM-003–1.5) showed a similar pattern to that of mdc-STM-001–0.6, with the benefit of 10-day HRs > 4 at an additional time point for the KIS colony (112 DAI).

### Median survival times

**Fig. 4:**
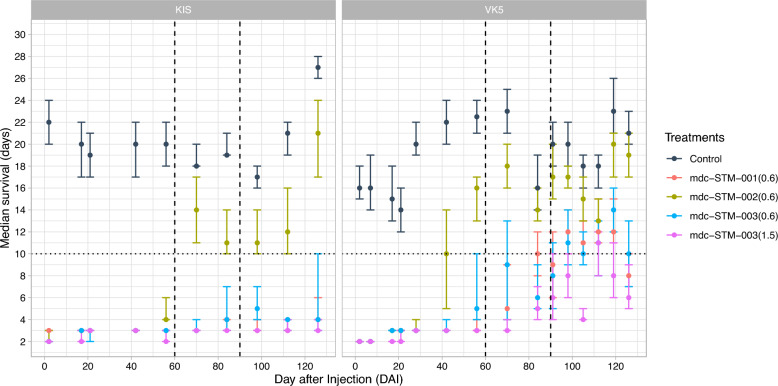
Median survival times of mosquitoes from both KIS and VK5 colonies fed at different days after injection of the LAIFs mdc-STM-001–0.6, mdc-STM-002–0.6, mdc-STM-003–06 and mdc-STM-003–1.5. The dashed horizontal line represents the average extrinsic incubation period for *Plasmodium falciparum*. The dashed vertical lines represent 2- and 3-month time points post-injection. Dots represent median values with the whiskers indicating the 95% confidence intervals. KIS,* Anopheles gambiae* colony Kisumu; LAIF, long-acting injectable ivermectin formulation; VK5,* Anopheles coluzzii* VK5 colony

Median survival times were consistently longer than 10 days for KIS and VK5 mosquitoes fed on control calves (Fig. 4) at all DAI time points tested.

For KIS mosquitoes, median survival times remained consistently < 10 days throughout the entire follow-up when fed on cattle treated with mdc-STM-001–0.6, mdc-STM-003–0.6 and mdc-STM-003–1.5. In contrast, mdc-STM-002–0.6 induced median survival times equal to or greater than the 10-day threshold from 70 DAI onward.

For VK5 mosquitoes, median survival times remained ≤ 10 days up to 91 days post-treatment, and again at DAI 126, when exposed to cattle treated with mdc-STM-001–0.6, mdc-STM-003–0.6, and mdc-STM-003–1.5 (Fig. 4). The LAIF mdc-STM-002–0.6 maintained median survival times below or equal to the EIP threshold only up to 40 DAI.

Notably, mdc-STM-001–0.6 and mdc-STM-003–1.5 induced particularly short survival durations, with median survival times < 4 days for nearly 100 days in KIS mosquitoes and approximately 60 days in VK5.

### Lethal concentrations and coverage duration

**Fig. 5:**
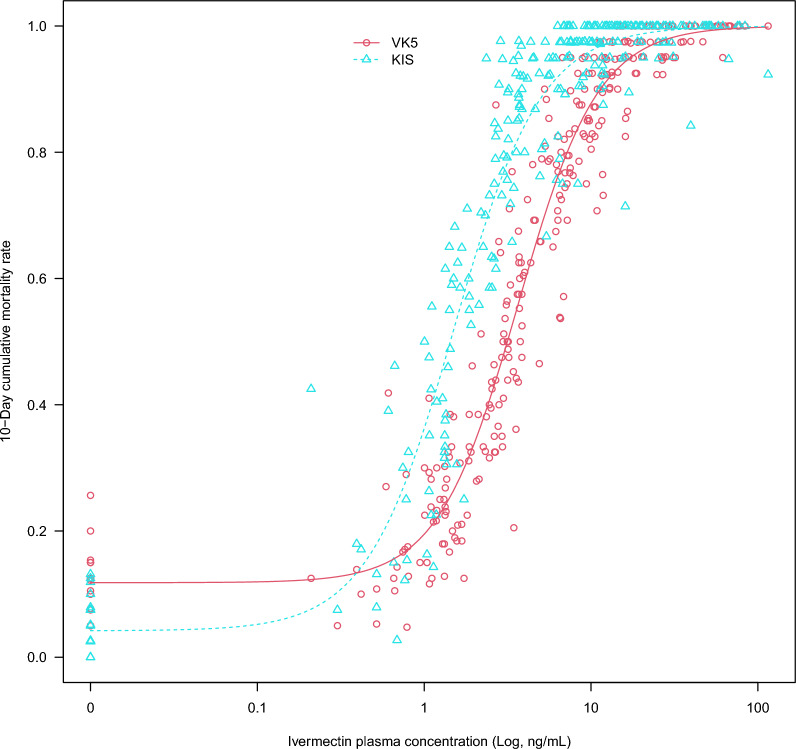
Relationship between ivermectin plasma concentrations and 10-day cumulative mosquito mortality for KIS and VK5 colonies. Data for 4-day cumulative mortality are shown in Additional file 2: Figure S6. KIS,* Anopheles gambiae* colony Kisumu; VK5,* Anopheles coluzzii* VK5 colony

Concentration/response relationships were examined for each colony and for each LAIF. No significant differences in lethal concentration 50 (LC50; concentration that kills 50% of the test population) values were found between the LAIFs tested, regardless of the mosquito colony (Additional file 1: Tables S9–S12; Additional file 2: Figures S4, and S5). A unified concentration–response model, including mosquito colony as explanatory variable, was thus developed using the full dataset (Fig. 5).

A significant difference in cumulative mortalities between the KIS and VK5 colonies was observed in relation to ivermectin concentrations, with 10-day LC50 values lower for the KIS test populations than for the VK5 test populations (mean LC50 for KIS = 1.51, 95% CI 1.1–2.07]; mean LC50 for VK5 3.66, 95% CI 2.69–4.97;* t*-value = − 6.3187, *P*-value < 0.0001). Ten-day LC50 values sufficient to kill KIS and VK5 mosquitoes were sustained for the whole experiment duration for KIS and for > 2 months for VK5, with the exception of the mdc-STM-002 formulation (Table 6). Values for the 4-day and 10-day LC50 and LC90 (lethal concentration 90) are given in Additional file 1: Table S13.

**Table 6:** Time period during which the LAIF candidates reach ivermectin plasma concentration values above or equal to 10-day LC50 for KIS and VK5 colonies

LAIF	KIS	VK5
mdc-STM-001-0.6	132 [132-132]	132 [67-132]
mdc-STM-002-0.6	73 [65-119]	50 [39-58]
mdc-STM-003-0.6	132 [132-132]	128 [78-132]
mdc-STM-003-1.5	132 [132-132]	132 [132-132]

Upper and lower confidence intervals (95%) are given in brackets

Inter-animal variability was also assessed, as it directly informs the coverage potential of each formulation, i.e. the proportion of treated animals maintaining mosquitocidal ivermectin plasma concentrations exceeding the LC50 over time. The mdc-STM-001–0.6 formulation achieved 100% coverage of VK5 10-day LC50 for a duration of at least 2 months post-dose (data not shown).

## Discussion

This study represents the first evaluation of candidate long-acting injectable formulations of ivermectin (LAIFs) specifically developed for human use in malaria control. These formulations are derived from unique long-lasting drug delivery technology and are designed to meet, with a single subcutaneous injection, the WHO’s minimum target product profile for endectocides against malaria, which is: achieving, with a single administration, at least 1 month of efficacy, defined by HR > 4 for the mortality of *Plasmodium* vectors. The primary objective of this work was to identify the most promising formulation for further development, including progression to phase 1 clinical trial. To guide formulation selection, we employed a comprehensive approach that integrated multiple, complementary criteria related to mosquitocidal efficacy, PK characteristics and inter-host variability, all parameters that are critical for achieving effective and sustained vector control in real-world conditions and settings. The mdc-STM-001 formulation met all of the predefined criteria and was selected as the optimal candidate for further evaluation in humans, based on the following key positive outcomes: (i) good local and systemic tolerability during and after injection, with no abnormal variation in body weight; (ii) optimal control of the initial burst, preventing systemic reactions during the early post-injection period; (iii) most suitable ivermectin PK and PD profiles, achieving LC50 levels over the target duration, resulting in the highest sustained mosquito mortality across both insecticide susceptible and resistant* Anopheles* strains; iv) the lowest inter-host variability in ivermectin exposure; and (v) appropriate clearance beyond the predefined release duration, avoiding unnecessary systemic exposure.

In terms of both magnitude and duration, the mosquitocidal efficacy of a single subcutaneous injection of the mdc-STM-001 formulation is compelling. It provides an efficacy duration of at least 2 months, which is at least fourfold longer than those achieved with either gastric-resident or conventional oral formulations, regardless of the dosing regimen considered. A sustained and high reduction in mosquito survival rate that lasts > 2 months is the factor expected to both most strongly and negatively influence vectorial capacity [53]. Furthermore, by consistently shortening mosquito lifespan below the 10-day EIP, the mdc-STM-001 formulation has strong potential to dramatically reduce the proportion of infectious mosquitoes and, consequently, malaria transmission across most epidemiological contexts.

### Strengths and limitations of the experimental model for extrapolation to humans

We acknowledge the limitation of extrapolating human PK and PD parameters from data obtained following cattle treatment with the LAIFs, as plasma concentration dynamics can vary substantially across species [54]. Because ivermectin is a highly lipophilic compound, body fat content, which also varies among species, represents a key determinant of its distribution within the organism [26]. Ivermectin tends to accumulate in adipose and dermal tissues surrounding the capillaries [55] from which mosquitoes obtain their blood meals. This may potentially lead to higher ivermectin concentrations being ingested by mosquitoes than those measured in venous plasma and used for PK/PD analyses [56]. Indeed, a previous clinical study reported ivermectin concentrations to be approximately 1.33-fold higher in capillary blood than in venous blood [57] even though this difference did not translate into measurable differences in mosquito mortality. In addition, cattle-derived secondary metabolites may also modulate the mosquitocidal effects of long-acting formulations, as has been suggested in humans [58] with possible species-specific differences in both the nature and kinetics of these metabolites (manuscript in preparation). For all of the above reasons, which are valid whatever the route of administration of ivermectin, extrapolation of PK and PD data from cattle to humans must be approached with caution, and venous plasma ivermectin concentrations should be regarded only as proxies for the actual levels of ivermectin and its metabolites ingested by mosquitoes. Nevertheless, it is noteworthy that in a phase 1 human trial using oral ivermectin, the venous plasma 10-day LC50 values lethal to *An. gambiae* sensu stricto fell within the same range as those obtained in the present study [26, 59].

Although our experimental cattle model, like any preclinical animal system, is subject to criticism regarding PK extrapolations to humans, it was deliberately selected to capture field-relevant *Anopheles gambiae * sensu lato fitness and ivermectin mosquitocidal effects since we used mosquito lots derived from a recently founded colony collected from Bobo Dioulasso surroundings. Indeed, throughout the Sudano-Sahelian zone, *Anopheles* mosquitoes frequently feed on domestic animals—particularly calves—which represent major blood-meal sources in areas of high bednet use [60, 61]. Mosquitoes feeding on calves exhibit survival rates and median lifespans comparable to those feeding on humans [62], reinforcing the biological relevance of this model. In addition, direct skin-feeding experiments on Burkinabé local calves are highly practical and result in consistently high feeding success, as these animals are natural alternative hosts for major malaria vectors. Our model therefore allows the proportion of blood-fed mosquitoes to be maximized and reflects their true post-feeding survival potential after feeding on treated or control hosts. Collectively, these parameters provide robust and field-transposable meaningful entomological endpoints that are essential for predicting the performance of the LAIFs in real-world settings.

### Mosquito resistance to ivermectin and mitigation plans

Resistance to ivermectin exists in different arthropods species [63, 64]. Similar to the oral formulation multiple dosing regimen, prolonged systemic exposure to ivermectin following treatment with the LAIF may exert selective pressure on resistance mechanisms, not only on its intended targets—human endoparasites—but also on *Anopheles* mosquitoes and other ectoparasites. Pyrethroids and ivermectin cross-resistance has been shown in permethrin-resistant *Aedes aegypti* colonies [65]. Given the distinct molecular targets of these two compound classes, the role of metabolic resistance pathways has been hypothesized. These mechanisms often involve the overexpression of detoxification enzymes, such as cytochrome P450s and xenobiotic efflux pumps [66, 67]. Supporting this, dual inhibition of P450s and xenobiotic transporters was found to significantly increase ivermectin susceptibility in *An. gambiae*, highlighting detoxification pathways as a credible resistance route [68]. Ivermectin resistance in *An. gambiae* could also arise due to existing alternative splicing of the gene coding for the GluCl channel, producing protein variants with differing sensitivities to ivermectin [69], which suggests that regulatory changes in splicing may contribute to reduced susceptibility.

Our findings reveal clear differences in ivermectin susceptibility between mosquito strains: the *An. coluzzii* VK5 colony required higher plasma lethal concentrations than the fully susceptible *An. gambiae* KIS strain, suggesting the presence of tolerance mechanisms in VK5. Previous studies have shown differences in susceptibility to ivermectin among different* Anopheles* species [22, 70, 71]. In the present study, we monitored the frequency of pyrethroid resistance alleles in the VK5 mosquito lots, which was high throughout the experiment; however, further individual-level characterization will be needed to determine whether a direct association, if any, exists between pyrethroid resistance genotypes and ivermectin tolerance in VK5. Beyond metabolic processes, factors such as distinct genetic backgrounds or morphological traits such as body size may contribute to an intertwined tolerance phenotype in which multiple mechanisms interact, making it difficult to disentangle their respective roles.

The risk of selecting ivermectin-resistant *Anopheles* populations during mass treatment campaigns does indeed exist, and could compromise sustained success of ivermectin-based malaria vector control strategies. In addition to physiological resistance, behavioral adaptations may further reduce the effectiveness of such interventions. Prolonged ivermectin exposure could result in selection for more zoophagic or opportunistic mosquito populations that preferentially feed on untreated animals or humans, thereby circumventing the drug’s effects and diminishing the overall intervention impact. Nonetheless, current MDA protocols for ivermectin include exclusion criteria that prevent the treatment of pregnant or lactating women and children under 15 kg body weight. Women of child bearing potential might further be excluded in the case of long-acting formulation deployment. These untreated subpopulations can act as genetic refuges, i.e. reservoirs of susceptible genotypes, which help maintain genetic diversity in vector and parasite populations and reduce the overall selection pressure for resistance [40, 72, 73]. Such refuges could contribute to the long-term sustainability of ivermectin-based interventions.

To preserve efficacy, it is critical that implementation strategies incorporate routine surveillance of both behavioral and physiological resistance [72]. However, there is currently no established and standardized assay for detecting ivermectin resistance in mosquitoes. While molecular markers will be instrumental in tracking the emergence and spread of resistance alleles, complementary phenotypic assays are urgently needed to detect functional resistance in the field. Traditional phenotypic methods rely on mosquitoes ingesting ivermectin via membrane feeding or direct feeding on treated hosts. These approaches, however, face practical limitations: membrane feeding often results in low blood uptake by mosquitoes, and direct feeding raises ethical concerns. A promising alternative involves delivering ivermectin-laced blood using filter papers [74].

### Coverage of blood sources

The residence time of ivermectin at efficient mosquitocidal concentrations in human blood is the principal determinant of its epidemiological impact, together with the proportion of the population exposed at such concentrations [75]. This has been demonstrated using modeling studies [30, 76]. The target population for standard oral ivermectin administration excludes pregnant women, nursing mothers and children with a body weight < 15 kg [77, 78], whereas the prolonged duration of action of mdc-STM-001 may also necessitate excluding women of child-bearing potential to avoid fetal exposure. Such restrictions would markedly reduce the target population size and could therefore limit overall effectiveness. However, models using simulated data from theoretical formulations with constant efficacy durations of 14 to 90 days demonstrate that, combined with seasonal malaria chemoprevention (SMC) strategies, longer lasting effects could lead to a significant decrease in malaria burden despite the reduced target population [79]. Further mathematical modeling, building on previous work [80], is ongoing using the present data to explore the potential of mdc-STM-001 under different implementation scenarios [81].

The specific safety concern regarding the inclusion of women of child-bearing potential is primarily related to possible fetal toxicity and is currently under debate [82], as is the inclusion of children weighing less than 15 kg [83]. Addressing these important matters will enable accurate projections for future study designs and implementation strategies.

As already mentioned earlier in this article, the effectiveness of long-acting ivermectin interventions will strongly depend on achieving sufficient coverage of the blood sources available to *Anopheles* mosquitoes, both human and animal. Detailed knowledge of mosquito bionomics within potential implementation zones is therefore critical to success [36], particularly since large regions of high malaria burden correspond to the Sudano-Sahelian belt, where vectors are highly opportunistic in their feeding behavior and often use domestic animals as alternative blood sources [60, 61]. In such settings, treating livestock with endectocidal compounds has been proposed as a promising complementary strategy [84]. Commercial veterinary injectable formulations have demonstrated efficacy for up to 1–2 months against Asian vectors in buffalo and cattle, respectively [70]. A veterinary prototype long-lasting formulation from the BEPO® technology extended this efficacy to more than 6 months for *An. coluzzii* [40], while silicone implants achieved comparable results [85]. Beyond enhancing the impact on vector populations, targeting both human and animal hosts could also contribute to mitigating the risk of ivermectin resistance if a distinct endectocide were used in animals.

### Acceptability and adherence

The deployment of a LAIF for malaria vector control would represent an innovative addition to the portfolio of community-based malaria interventions. Similar to other public health strategies relying on high population coverage—such as vaccination programs, anti-helminthic MDAs or malaria vector control campaigns—its success will depend on strong acceptability and adherence. Conceptually aligned with transmission-blocking vaccines (TBVs), the LAIF acts primarily through community protection. Regarding the molecule, evidence from decades of oral ivermectin MDAs for filarial control demonstrates high community acceptability—often > 90%—although adherence tends to vary across rounds [86]. The same trend has been observed when ivermectin is proposed as a vector control tool against malaria [87]. Similarly, the four-dose injectable vaccine RTS,S/AS01 has achieved strong community acceptance (80–95%) when supported by effective communication and community engagement [88, 89]. Acceptability studies for TBV concepts have also reported willingness- to-receive rates > 90% in high-burden settings [90]. For the LAIF, the anticipated subcutaneous injection volumes (up to a maximum of 25 µl/kg) is relatively small, suggesting minimal practical or acceptability constraints for large-scale implementation. Importantly, unlike TBVs, an LAIF intervention could also provide direct individual health benefits by preventing or treating parasitic infections traditionally controlled by ivermectin, while simultaneously reducing malaria transmission. Additional collateral benefits from ivermectin MDAs against malaria have recently been reported, including reductions in childhood anemia [35] and decreases in jigger infestations and bedbug nuisances [91]. This dual individual and community benefit may substantially enhance perceived value, acceptability and adherence compared to TBVs. By combining infrequent dosing, a familiar and trusted product with a well-known safety profile [92] and a clear community rationale, ivermectin MDA using LAIF could become a socially acceptable and operationally feasible complement to existing malaria control and vaccination platforms. Furthermore, such a strategy could leverage the logistical infrastructure and community trust established through vaccination programs, facilitating delivery, adherence monitoring, and social acceptance.

## Conclusions

This study demonstrates that among the three long-acting injectable ivermectin formulations developed for human use, the mdc-STM-001 formulation exhibits the most favorable combination of PK properties, PD efficacy and tolerability in a cattle–mosquito model. A single subcutaneous administration of mdc-STM-001 achieved sustained mosquitocidal concentrations over 2 months, inducing high mortality rate in both pyrethroid-susceptible and -resistant* Anopheles* strains, thereby meeting and exceeding the WHO-defined minimum criteria for endectocides targeting malaria vectors. These findings support the potential of mdc-STM-001 as a promising tool to complement existing vector control strategies, particularly in areas with behavioral or physiological resistance to conventional insecticides. While extrapolation from cattle to humans warrants caution, the observed pharmacological profile and robust entomological endpoints provide a strong rationale for advancing this formulation into phase 1 clinical trials. Safety will remain a key consideration given the long-lasting systemic exposure, although the multiple administrations of high oral dose are safe, which supports a favorable risk–benefit perspective for further human evaluation. The formulation will also retain the positive collateral benefits observed with oral ivermectin, such as reductions in parasitic infections, childhood anemia and ectoparasite nuisances, underscoring the value of mdc-STM-001 as both a community- and individual-level intervention, potentially further enhancing community acceptability and adherence. Future investigations should leverage modeling approaches to optimize dosing regimens and explore synergistic integration with other vector control strategies, including seasonal malaria chemoprevention, LLINs, indoor residual spraying or livestock-targeted endectocide interventions. Such models will also help anticipate the population coverage and logistical requirements needed to maximize epidemiological impact while ensuring operational feasibility. Given the potential for physiological or behavioral resistance to emerge, continued studies on mitigating resistance dynamics in vector populations will be essential to ensure sustainable efficacy. Overall, ivermectin MDA using the mdc-STM-001 long-acting injectable formulation based on BEPO^®^ technology represents a potentially transformative adjunct to existing malaria control measures, with the capacity to reduce transmission by targeting both indoor- and outdoor-biting vectors over extended periods.

## Supplementary Information

Additional file 1: Table S1. Injected volumes (ml) per animal and per formulation. Table S2. Median weight (Q1, Q3) in kg for each experimental cattle group at each weighing date. Table S3. Additional mean (SD) pharmacokinetic parameters of ivermectin in cattle plasma. Table S4. Ivermectin extraction results in each depot collected at study end. Table S5. Copolymers extraction results in each depot collected at study end. Table S6. Number of mosquitoes homozygous or heterozygous for the mutated KDR-W allele. Table S7. Number of KIS (**A**) and VK5 (**B**) blood-fed mosquitoes followed for their survival throughout the experiment. Table S8. Hazard ratios (HRs),* z*-values and associated* p*-values derived from the Cox proportional hazards models for KIS and VK5 mosquitoes. Tables S9–S12. Comparison of the lethal concentrations of formulations. Table S13. Lethal concentration (LC50) for KIS and VK5 mosquitoes over 4-day follow-up period (4-day LC50)

Additional file 2: Figure S1. Additional mean concentration–time profiles of ivermectin in cattle plasma. Figure S2. Kaplan-Meier plots. Figure S3. Four-day cumulative mortality of KIS and VK5. Figure S4. Heat maps illustrating the lower 95% confidence limit (LCL) of 4-day and 30-day mortality hazard ratios (HRs) for KIS and VK5 colony mosquitoes. Figure S5. Relationship between ivermectin plasma concentration and 4-day (**A**, **B**) or 10-day (**C **,** D**) cumulative mortality for each candidate formulation. Figure S6. Relationship between ivermectin plasma concentrations and 4-day cumulative mosquito mortality for KIS and VK5 colonies

## Data Availability

The datasets and codes used for the analyses and figure generation reported in this study are publicly available at 10.23708/PSJMLM

## Abbreviations

CIRDES: Centre International de Recherche–Développement sur l’Élevage en zone Subhumide
DAI: Day(s) after injection
IRSS: Institut de Recherche en Sciences de la Santé
K2-EDTA: Dipotassium ethylenediaminetetraacetic acid
KDR: Knockdown resistance
LAIF: Long-acting ivermectin formulation
LC50: Lethal concentration 50
LC–MS/MS: Liquid chromatography–tandem mass spectrometry
MDA: Mass drug administration
PD: Pharmacodynamic
PK: Pharmacokinetic
SMC: Seasonal malaria chemoprevention
TBV: Transmission-blocking vaccines
