# Supplementary material for: Selection of the optimal long-acting injectable formulation of ivermectin for use in humans to target malaria vectors in Western Africa: evaluation of pharmacokinetics and mosquitocidal efficacy in cattle under laboratory conditions
Source: Parasit Vectors. 2026 May 6;19:226. doi: 10.1186/s13071-026-07263-x (PMC13192019; doi:10.1186/s13071-026-07263-x)
Supplement: Supplementary file 1 — Additional file 1: Table S1. Injected volumes (ml) per animal and per formulation. Table S2. Median weight (Q1, Q3) in kg for each experimental cattle group at each weighing date. Table S3. Additional mean (SD) pharmacokinetic parameters of ivermectin in cattle plasma. Table S4. Ivermectin extraction results in each depot collected at study end. Table S5. Copolymers extraction results in each depot collected at study end. Table S6. Number of mosquitoes homozygous or heterozygous for the mutated KDR-W allele. Table S7. Number of KIS (A) and VK5 (B) blood-fed mosquitoes followed for their survival throughout the experiment. Table S8. Hazard ratios (HRs), z-values and associated p-values derived from the Cox proportional hazards models for KIS and VK5 mosquitoes. Tables S9–S12. Comparison of the lethal concentrations of formulations. Table S13. Lethal concentration (LC50) for KIS and VK5 mosquitoes over 4-day follow-up period (4-day LC50) [file 13071_2026_7263_MOESM1_ESM.docx]

### Supplementary Table S1. Injected volumes (mL) per animal and per formulation, given the body weight (BW) measured the day of the treatment (kg)

| Animal ID | BW at arrival (kg) | BW at injection (kg) | Formulation ID | Injected volume (mL) |
| --- | --- | --- | --- | --- |
| B122 | 141.6 | 153.4 | mdc-STM-001 | 1.18 |
| B126 | 116.8 | 140.2 | mdc-STM-001 | 1.08 |
| B128 | 109.4 | 119.8 | mdc-STM-001 | 0.92 |
| B332 | 119.0 | 126.6 | mdc-STM-001 | 0.98 |
| B335 | 99.2 | 115.2 | mdc-STM-001 | 0.89 |
| B121 | 107.2 | 120.8 | mdc-STM-002 | 0.94 |
| B130 | 128.8 | 137.6 | mdc-STM-002 | 1.07 |
| B324 | 97.4 | 115.2 | mdc-STM-002 | 0.90 |
| B330 | 138.0 | 144.6 | mdc-STM-002 | 1.13 |
| B334 | 142.0 | 151.4 | mdc-STM-002 | 1.18 |
| B123 | 101.2 | 122.3 | mdc-STM-003 | 0.72 |
| B124 | 124.4 | 130.6 | mdc-STM-003 | 0.76 |
| B125 | 124.6 | 142.2 | mdc-STM-003 | 0.83 |
| B326 | 140.8 | 160.8 | mdc-STM-003 | 0.94 |
| B333 | 94.2 | 106.6 | mdc-STM-003 | 0.62 |
| B322 | 127.4 | 146.8 | mdc-STM-003 | 2.14 |
| B323 | 108.6 | 120.0 | mdc-STM-003 | 1.75 |
| B327 | 129.4 | 144.6 | mdc-STM-003 | 2.11 |
| B329 | 99.8 | 115.8 | mdc-STM-003 | 1.69 |
| B331 | 106.4 | 124.8 | mdc-STM-003 | 1.82 |

### Supplementary Table S2. Median weight (Q1, Q3) in kg for each experimental cattle group at each weighing date.

| Experimental arm | Control  N=5 | mdc-STM-001  N=5 | mdc-STM-002  N=5 | mdc-STM-003  N=5 | mdc-STM-004  N=5 |
| --- | --- | --- | --- | --- | --- |
| Weighing date |  |  |  |  |  |
| 2020-11-25 | 114 (110,125) | 117 (109,119) | 129 (107,138) | 124 (101,125) | 109 (106,127) |
| 2020-12-24 | 116 (116,126) | 118 (114, 123) | 134 (105,136) | 121 (109,126) | 113 (112,133) |
| 2021-01-14 | 129 (123,140) | 127 (120,140) | 138 (121,145) | 131 (122, 142) | 125 (120, 145) |
| 2021-02-15 | 138 (138,151) | 135 (134,153) | 157 (134,162) | 146 (138,159) | 139 (134,157) |
| 2021-03-15 | 144 (141,152) | 141 (141,158) | 164 (134,169) | 150 (138,162) | 142 (140,163) |
| 2021-04-15 | 162 (155,162) | 160 (152,170) | 177 (149,178) | 166 (148,181) | 163 (149,178) |
| 2021-05-15 | 161 (155,167) | 164 (161,177) | 183 (157,186) | 167 (158,182) | 170 (149,173) |

### Supplementary Table S3. Mean (SD) pharmacokinetic parameters of ivermectin in cattle plasma after single subcutaneous administration of LAIFs at 0.6 or 1.5 mg/kg

| Parameters | mdc-STM-001-0.6 | mdc-STM-002-0.6 | mdc-STM-003-0.6 | mdc-STM-003-1.5 |
| --- | --- | --- | --- | --- |
| Tmax _a_ (h) | 24 (24; 48) | 48 (24; 168) | 24 (24; 48) | 24 (24; 168) |
| Cmax (ng/mL) | 35.4 (12.7) | 50.3 (13.7) | 58.1 (25.2) | 81.2 (32.2) |
| Cmax/Dose (ng/mL/mg/kg) | 58.3 (22.0) | 84.3 (22.6) | 99.4 (42.2) | 56.1 (22.4) |
| Clast (ng/mL) | 3.93 (2.75) | 1.03 (0.70) | 3.43 (2.74) | 6.30 (4.35) |
| AUC(0-28d) (h.ng/mL) | 14227 (3853) | 16253 (5465) | 18085 (9789) | 26248 (5534) |
| AUC(0-28d)/Dose  (h.ng/mL/mg/kg) | 23378 (6862) | 27298 (9171) | 30956 (16689) | 18116 (3822) |
| AUC(0-91d) (h.ng/mL) | 25055 (2936) | 21121 (8866) | 28890 (18668) | 41820 (13286) |
| AUC(0-91d)/Dose  (h.ng/mL/mg/kg) | 40990 (5664) | 35479 (14839) | 49483 (31994) | 28903 (9300) |
| AUClast (h.ng/mL) | 28534 (2316) | 22322 (9059) | 32851 (21691) | 47192 (15724) |
| AUClast/Dose  (h.ng/mL/mg/kg) | 46589 (3960) | 37497 (15165) | 56269 (37147) | 32630 (11042) |
| T1/2 (h) | 784 (244) (n=4) | 731 (579) (n=4) | 728 (399) (n=3) | 489 (n=1) |
| C28d (ng/mL) | 13.7 (1.6) | 8.90 (4.85) | 11.1 (8.3) | 15.5 (5.1) |
| C56d (ng/mL) | 6.70 (1.67) | 2.92 (2.63) | 7.34 (7.14) | 10.3 (6.9) |
| C91d (ng/mL) | 3.48 (1.85) | 1.11 (0.84) | 4.33 (3.99) | 5.55 (3.72) |

SD: standard deviation.

_a_ Median (min; max).

Tmax: time to reach Cmax. Cmax: maximal plasma concentration. Clast: last quantifiable plasma concentration. AUC(0-28d): area under curve from time 0 to 28 days. AUC(0-28d)/Dose: dose-normalized AUC(0-28d). AUC(0-91d): area under curve from time 0 to 91 days. AUC(0-91d)/Dose: dose-normalized AUC(0-91d). AUClast: area under curve from time 0 to last quantifiable plasma level. AUClast/Dose: dose-normalized AUClast. T1/2: apparent terminal phase half-life. C28d: plasma concentration 28 days post injection. C56d: plasma concentration 56 days post injection. C91d: plasma concentration 91 days post injection.

### Supplementary Table S4. Ivermectin extraction results in each depot collected at study end

| Treatment  Dose (mg/kg) | Animal ID | IVM injected dose (mg) | IVM recovered dose (mg) | | IVM recovered dose (%) | |
| --- | --- | --- | --- | --- | --- | --- |
|  |  |  | Indiv. | Mean (SD) | Indiv. | Mean (SD) |
| mdc-STM-001-0.6 | B122 | 100.4 | 38.6 | 19.4 (12.9) | 38.5 | 23.0 (12.7) |
|  | B126 | 85.0 | 18.3 |  | 21.6 |  |
|  | B128 | 73.3 | 2.5 |  | 3.3 |  |
|  | B332 | 76.3 | 20.6 |  | 27.0 |  |
|  | B335 | 67.9 | 16.8 |  | 24.8 |  |
| mdc-STM-002-0.6 | B121 | 76.1 | 39.0 | 32.3 (17.7) | 51.3 | 39.1 (19.6) |
|  | B130 | 84.7 | 6.1 |  | 7.2 |  |
|  | B324 | 69.0 | 23.4 |  | 33.9 |  |
|  | B330 | 87.7 | 41.7 |  | 47.6 |  |
|  | B334 | 92.0 | 51.1 |  | 55.5 |  |
| mdc-STM-003-0.6 | B123 | 70.6 | 5.4 | 17.8 (14.4) | 7.7 | 23.1 (17.6) |
|  | B124 | 76.4 | 14.3 |  | 18.7 |  |
|  | B125 | 81.1 | 42.6 |  | 52.6 |  |
|  | B326 | 96.4 | 12.0 |  | 12.5 |  |
|  | B333 | 61.6 | 14.8 |  | 24.0 |  |
| mdc-STM-003-1.5 | 127.4 | 211.6 | 69.8 | 59.5 (35.5) | 33.0 | 32.7 (20.0) |
|  | 108.6 | 173.8 | 55.5 |  | 32.0 |  |
|  | 129.4 | 212.3 | 0.5 |  | 0.2 |  |
|  | 99.8 | 164.8 | 79.7 |  | 48.4 |  |
|  | 106.4 | 182.9 | 91.8 |  | 50.2 |  |

IVM: ivermectin. Indiv.: individual. SD: standard deviation.

### Supplementary Table S5. Copolymers extraction results in each depot collected at study end

| Treatment  Dose (mg/kg) | Animal ID | Copolymer recovered  (%) | |
| --- | --- | --- | --- |
|  |  | Indiv. | Mean (SD) |
| mdc-STM-001-0.6 | B122 | 27.2 | 18.8 (8.5) |
|  | B126 | 24.0 |  |
|  | B128 | 5.4 |  |
|  | B332 | 16.5 |  |
|  | B335 | 21.1 |  |
| mdc-STM-002-0.6 | B121 | 48.6 | 34.1 (15.9) |
|  | B130 | 8.0 |  |
|  | B324 | 39.2 |  |
|  | B330 | 43.4 |  |
|  | B334 | 31.0 |  |
| mdc-STM-003-0.6 | B123 | 16.1 | 25.9 (25.0) |
|  | B124 | 6.8 |  |
|  | B125 | 22.1 |  |
|  | B326 | 15.1 |  |
|  | B333 | 69.5 |  |
| mdc-STM-003-1.5 | 127.4 | 42.1 | 34.1 (18.8) |
|  | 108.6 | 42.9 |  |
|  | 129.4 | 0.8 |  |
|  | 99.8 | 46.1 |  |
|  | 106.4 | 38.6 |  |

Indiv.: individual. SD: standard deviation.

Supplementary Table S6. Number of mosquitoes homozygous or heterozygous for the mutated KDR-W allele (resistant allele, R) of the voltage-gated sodium channel gene, which confers resistance to pyrethroids. In contrast, the wild-type allele is designated S (susceptible allele). RR and RS indicate homozygous and heterozygous genotypes for the KDR-W mutation, respectively. SS corresponds to homozygosity for the wild-type allele. Resistant phenotypes (RR and RS genotypes) among the tested mosquitoes (n) are given as percentages. Numbers are reported per generation (F2 to F9) and per day after injection (DAI) at which mosquitoes were exposed to cattle.

| DAI | Generation (F) | RR (n) | RS (n) | SS (n) | Resistant phenotypes (%) | Moquitoes tested (n) |
| --- | --- | --- | --- | --- | --- | --- |
| pre-dose | F2 | 10 | 15 | 8 | 73,52 | 33 |
| 2, 7, 17, 21, 28 | F3 | 2 | 6 | 2 | 80 | 10 |
| 42 | F5 | 12 | 20 | 9 | 74,41 | 41 |
| 56 | F6 |  |  |  | na | na |
| 70, 84, 91, 98 | F7 | 6 | 19 | 21 | 54,34 | 47 |
| 105,112,119 | F8 |  |  |  | na | na |
| 126 | F9 | 5 | 14 | 17 | 52,77 | 36 |

Supplementary Table S7A. Number of KIS blood fed mosquitoes followed for their survival before injection (BI) and at the different days after injection (DAI) for each cattle of the different treatment groups.

|  |  |  |  | Day after injection (DAI) | | | | | | | | | | | | | | |
| --- | --- | --- | --- | --- | --- | --- | --- | --- | --- | --- | --- | --- | --- | --- | --- | --- | --- | --- |
| Mosquito Colony | Treatment | Cattle ID | BI | 2 | 7 | 17 | 21 | 28 | 42 | 56 | 70 | 84 | 91 | 98 | 105 | 112 | 119 | 126 |
| kis | Control | B127 | 40 | 31 | 0 | 37 | 39 | 0 | 19 | 29 | 41 | 40 | 0 | 40 | 37 | 38 | 39 | 39 |
|  |  | B129 | 40 | 34 | 0 | 31 | 41 | 0 | 38 | 31 | 41 | 41 | 0 | 36 | 32 | 38 | 40 | 40 |
|  |  | B321 | 40 | 34 | 0 | 38 | 34 | 0 | 39 | 38 | 41 | 40 | 0 | 0 | 0 | 39 | 43 | 41 |
|  |  | B328 | 41 | 20 | 0 | 35 | 37 | 0 | 32 | 38 | 42 | 40 | 0 | 42 | 42 | 41 | 41 | 38 |
|  |  | B336 | 38 | 34 | 0 | 37 | 42 | 0 | 19 | 32 | 41 | 40 | 0 | 39 | 39 | 41 | 39 | 40 |
|  | mdc-STM-001-0.6 | B122 | 40 | 38 | 0 | 39 | 40 | 20 | 40 | 37 | 40 | 39 | 0 | 40 | 37 | 40 | 41 | 42 |
|  |  | B126 | 39 | 40 | 0 | 40 | 40 | 21 | 40 | 38 | 38 | 36 | 0 | 40 | 40 | 42 | 38 | 36 |
|  |  | B128 | 38 | 28 | 0 | 39 | 41 | 9 | 40 | 41 | 41 | 40 | 0 | 0 | 0 | 40 | 37 | 41 |
|  |  | B332 | 40 | 41 | 0 | 34 | 39 | 21 | 38 | 42 | 40 | 44 | 0 | 38 | 40 | 43 | 39 | 38 |
|  |  | B335 | 40 | 38 | 0 | 40 | 40 | 24 | 32 | 39 | 39 | 43 | 0 | 39 | 41 | 41 | 40 | 41 |
|  | mdc-STM-002-0.6 | B121 | 38 | 38 | 0 | 40 | 43 | 10 | 37 | 44 | 38 | 40 | 0 | 39 | 36 | 41 | 40 | 40 |
|  |  | B130 | 40 | 39 | 0 | 40 | 40 | 21 | 40 | 42 | 32 | 40 | 0 | 38 | 15 | 39 | 38 | 39 |
|  |  | B324 | 40 | 39 | 0 | 40 | 40 | 21 | 40 | 39 | 40 | 40 | 0 | 37 | 40 | 36 | 39 | 37 |
|  |  | B330 | 40 | 41 | 0 | 41 | 40 | 24 | 31 | 40 | 39 | 40 | 0 | 40 | 38 | 39 | 39 | 39 |
|  |  | B334 | 40 | 38 | 0 | 40 | 40 | 18 | 40 | 38 | 39 | 43 | 0 | 0 | 0 | 42 | 40 | 38 |
|  | mdc-STM-003-0.6 | B123 | 40 | 40 | 0 | 41 | 38 | 10 | 40 | 40 | 40 | 39 | 0 | 38 | 37 | 40 | 39 | 38 |
|  |  | B124 | 38 | 39 | 0 | 37 | 39 | 6 | 40 | 40 | 40 | 42 | 0 | 39 | 42 | 38 | 37 | 40 |
|  |  | B125 | 40 | 40 | 0 | 38 | 40 | 25 | 39 | 38 | 40 | 41 | 0 | 0 | 0 | 38 | 39 | 41 |
|  |  | B326 | 40 | 37 | 0 | 39 | 40 | 16 | 40 | 38 | 40 | 42 | 0 | 39 | 37 | 39 | 40 | 40 |
|  |  | B333 | 40 | 42 | 0 | 39 | 39 | 4 | 41 | 36 | 43 | 38 | 0 | 40 | 33 | 40 | 40 | 42 |
|  | mdc-STM-003-1.5 | B322 | 40 | 39 | 0 | 39 | 40 | 20 | 40 | 39 | 40 | 42 | 0 | 41 | 37 | 38 | 42 | 40 |
|  |  | B323 | 40 | 40 | 0 | 39 | 40 | 18 | 39 | 32 | 40 | 40 | 0 | 25 | 37 | 40 | 42 | 44 |
|  |  | B327 | 40 | 35 | 0 | 38 | 40 | 17 | 41 | 24 | 38 | 41 | 0 | 43 | 26 | 36 | 39 | 41 |
|  |  | B329 | 38 | 38 | 0 | 40 | 40 | 0 | 35 | 40 | 41 | 42 | 0 | 41 | 36 | 40 | 41 | 39 |
|  |  | B331 | 40 | 38 | 0 | 38 | 40 | 6 | 30 | 39 | 39 | 40 | 0 | 0 | 0 | 42 | 38 | 39 |
| Total |  |  | 990 | 921 | 0 | 959 | 992 | 311 | 910 | 934 | 993 | 1013 | 0 | 774 | 722 | 991 | 990 | 993 |

Supplementary Table S7B. Number of VK5 blood fed mosquitoes followed for their survival before injection (BI) and at the different days after injection (DAI) for each cattle of the different treatment groups.

|  |  |  | | |  | Days after injection (DAI) | | | | | | | | | | | | | | | | | |  |
| --- | --- | --- | --- | --- | --- | --- | --- | --- | --- | --- | --- | --- | --- | --- | --- | --- | --- | --- | --- | --- | --- | --- | --- | --- |
| Mosquito Colony | Treatment | | Cattle ID | BI | | 2 | 7 | 17 | 21 | 28 | 42 | | 56 | 70 | 84 | 91 | 98 | 105 | 112 | 119 | | 126 | | |
| vk5 | Control | | B127 | 40 | | 41 | 31 | 39 | 41 | 38 | 39 | | 40 | 38 | 40 | 42 | 40 | 41 | 39 | | 40 | | 41 | |
|  |  |  | B129 | 40 | | 39 | 34 | 40 | 40 | 39 | 40 | | 39 | 41 | 41 | 41 | 40 | 41 | 38 | | 39 | | 39 | |
|  |  |  | B321 | 40 | | 38 | 36 | 42 | 34 | 36 | 40 | | 41 | 37 | 40 | 39 | 41 | 40 | 39 | | 39 | | 38 | |
|  |  |  | B328 | 40 | | 40 | 38 | 38 | 39 | 37 | | 40 | 42 | 40 | 42 | 39 | 42 | 41 | 39 | | 40 | | 41 | |
|  |  |  | B336 | 41 | | 37 | 39 | 36 | 37 | 41 | | 40 | 42 | 37 | 42 | 42 | 43 | 42 | 40 | | 41 | | 42 | |
|  | mdc-STM-001-0.6 | | B122 | 40 | | 41 | 39 | 40 | 40 | 41 | | 40 | 40 | 40 | 41 | 40 | 39 | 43 | 41 | | 42 | | 41 | |
|  |  |  | B126 | 40 | | 41 | 40 | 39 | 41 | 40 | | 39 | 39 | 39 | 42 | 40 | 40 | 43 | 40 | | 40 | | 40 | |
|  |  |  | B128 | 39 | | 39 | 40 | 39 | 40 | 38 | | 41 | 41 | 39 | 41 | 40 | 40 | 40 | 39 | | 42 | | 41 | |
|  |  |  | B332 | 40 | | 40 | 37 | 40 | 40 | 38 | | 41 | 41 | 40 | 40 | 38 | 39 | 41 | 41 | | 42 | | 39 | |
|  |  |  | B335 | 41 | | 41 | 40 | 39 | 41 | 41 | | 40 | 38 | 38 | 40 | 40 | 42 | 40 | 38 | | 40 | | 40 | |
|  | mdc-STM-002-0.6 | | B121 | 38 | | 41 | 40 | 41 | 40 | 40 | | 38 | 37 | 38 | 38 | 37 | 38 | 40 | 39 | | 42 | | 42 | |
|  |  |  | B130 | 40 | | 38 | 37 | 40 | 40 | 41 | | 38 | 39 | 40 | 46 | 39 | 40 | 45 | 39 | | 39 | | 39 | |
|  |  |  | B324 | 40 | | 40 | 40 | 40 | 40 | 42 | | 39 | 41 | 39 | 42 | 40 | 43 | 42 | 39 | | 39 | | 40 | |
|  |  |  | B330 | 40 | | 39 | 39 | 40 | 39 | 40 | | 37 | 40 | 36 | 40 | 40 | 38 | 43 | 40 | | 43 | | 37 | |
|  |  |  | B334 | 40 | | 39 | 41 | 41 | 40 | 38 | | 40 | 40 | 40 | 39 | 38 | 39 | 43 | 39 | | 43 | | 40 | |
|  | mdc-STM-003-0.6 | | B123 | 40 | | 40 | 40 | 40 | 39 | 39 | | 39 | 40 | 40 | 40 | 40 | 41 | 41 | 42 | | 40 | | 43 | |
|  |  |  | B124 | 39 | | 40 | 39 | 40 | 39 | 43 | | 36 | 41 | 39 | 42 | 40 | 41 | 43 | 40 | | 43 | | 39 | |
|  |  |  | B125 | 40 | | 39 | 38 | 42 | 42 | 41 | | 39 | 40 | 40 | 40 | 41 | 39 | 43 | 41 | | 40 | | 41 | |
|  |  |  | B326 | 40 | | 40 | 39 | 40 | 41 | 34 | | 40 | 39 | 40 | 40 | 40 | 43 | 43 | 39 | | 40 | | 39 | |
|  |  |  | B333 | 40 | | 40 | 41 | 40 | 38 | 40 | | 38 | 38 | 40 | 39 | 40 | 40 | 42 | 41 | | 40 | | 39 | |
|  | mdc-STM-003-1.5 | | B322 | 40 | | 39 | 40 | 40 | 40 | 41 | | 39 | 39 | 40 | 38 | 38 | 40 | 40 | 40 | | 39 | | 39 | |
|  |  |  | B323 | 39 | | 39 | 39 | 38 | 39 | 37 | | 41 | 40 | 40 | 40 | 41 | 43 | 40 | 43 | | 39 | | 41 | |
|  |  |  | B327 | 40 | | 40 | 40 | 40 | 40 | 39 | | 38 | 39 | 37 | 43 | 40 | 39 | 40 | 40 | | 42 | | 40 | |
|  |  |  | B329 | 40 | | 41 | 40 | 39 | 39 | 37 | | 37 | 40 | 41 | 40 | 40 | 39 | 40 | 41 | | 43 | | 41 | |
|  |  |  | B331 | 40 | | 40 | 39 | 40 | 39 | 41 | | 39 | 41 | 40 | 39 | 40 | 38 | 41 | 39 | | 41 | | 39 | |
| Total |  | |  | 997 | | 992 | 966 | 993 | 988 | 982 | | 978 | 997 | 979 | 1015 | 995 | 1007 | 1038 | 996 | | 1018 | | 1001 | |

### Supplementary table S8. Hazard ratios (HRs), z-values, and associated p-values derived from the Cox proportional hazards models. Values are given for 4, 10 and 30-days of survival follow-up after mosquitoes blood feeding, before injection (BI) and for each day after injection (DAI). A. KIS mosquitoes. B. VK5 mosquitoes.

**A.**

| Formulation | Treatment arm | DAI | Follow-up  (days) | HR (CI 95%) | z.ratio | p.value |
| --- | --- | --- | --- | --- | --- | --- |
| (MDC-STM-001-0.6) | Control | BI | 4 | 1 (0.1-17.2) | 0.0018088 | 1.0000000 |
| (MDC-STM-002-0.6) | Control | BI | 4 | 1.9 (0.2-22.7) | 0.6900508 | 0.9587058 |
| (MDC-STM-003-0.6) | Control | BI | 4 | 0.8 (0-14.3) | -0.1754490 | 0.9997884 |
| (MDC-STM-003-1.5) | Control | BI | 4 | 1.9 (0.2-22.4) | 0.6751535 | 0.9618095 |
| (MDC-STM-001-0.6) | Control | 2 | 4 | 32.2 (9.8-106.3) | 7.9378105 | 0.0000000 |
| (MDC-STM-002-0.6) | Control | 2 | 4 | 59.3 (18-195.1) | 9.3486912 | 0.0000000 |
| (MDC-STM-003-0.6) | Control | 2 | 4 | 58.3 (17.7-191.8) | 9.3098183 | 0.0000000 |
| (MDC-STM-003-1.5) | Control | 2 | 4 | 57.3 (17.4-188.9) | 9.2598711 | 0.0000000 |
| (MDC-STM-001-0.6) | Control | 17 | 4 | 56 (17-184.3) | 9.2185509 | 0.0000000 |
| (MDC-STM-002-0.6) | Control | 17 | 4 | 53.4 (16.2-175.7) | 9.1132259 | 0.0000000 |
| (MDC-STM-003-0.6) | Control | 17 | 4 | 50.4 (15.3-165.8) | 8.9748191 | 0.0000000 |
| (MDC-STM-003-1.5) | Control | 17 | 4 | 60.6 (18.4-199.5) | 9.3992086 | 0.0000000 |
| (MDC-STM-001-0.6) | Control | 21 | 4 | 25.5 (9.3-70.3) | 8.7248194 | 0.0000000 |
| (MDC-STM-002-0.6) | Control | 21 | 4 | 23.4 (8.5-64.4) | 8.4758112 | 0.0000000 |
| (MDC-STM-003-0.6) | Control | 21 | 4 | 27.7 (10.1-76.4) | 8.9365409 | 0.0000000 |
| (MDC-STM-003-1.5) | Control | 21 | 4 | 27.7 (10-76.2) | 8.9388090 | 0.0000000 |
| (MDC-STM-001-0.6) | Control | 42 | 4 | 41.1 (10.2-165.9) | 7.2625835 | 0.0000000 |
| (MDC-STM-002-0.6) | Control | 42 | 4 | 24 (5.9-97.5) | 6.1919836 | 0.0000000 |
| (MDC-STM-003-0.6) | Control | 42 | 4 | 28.2 (7-114.1) | 6.5248654 | 0.0000000 |
| (MDC-STM-003-1.5) | Control | 42 | 4 | 40.3 (10-162.9) | 7.2196770 | 0.0000000 |
| (MDC-STM-001-0.6) | Control | 56 | 4 | 55.9 (14-223.3) | 7.9184506 | 0.0000000 |
| (MDC-STM-002-0.6) | Control | 56 | 4 | 23.6 (5.8-95) | 6.1826656 | 0.0000000 |
| (MDC-STM-003-0.6) | Control | 56 | 4 | 52.2 (13-209.7) | 7.7629011 | 0.0000000 |
| (MDC-STM-003-1.5) | Control | 56 | 4 | 81.4 (20.3-326.2) | 8.6502114 | 0.0000000 |
| (MDC-STM-001-0.6) | Control | 70 | 4 | 26.4 (8.2-84.9) | 7.6620884 | 0.0000000 |
| (MDC-STM-002-0.6) | Control | 70 | 4 | 6.2 (1.9-20.7) | 4.1257303 | 0.0003562 |
| (MDC-STM-003-0.6) | Control | 70 | 4 | 19.8 (6.1-64) | 6.9522992 | 0.0000000 |
| (MDC-STM-003-1.5) | Control | 70 | 4 | 34.4 (10.7-110.5) | 8.2674788 | 0.0000000 |
| (MDC-STM-001-0.6) | Control | 84 | 4 | 7.9 (2.9-21.6) | 5.5987636 | 0.0000002 |
| (MDC-STM-002-0.6) | Control | 84 | 4 | 2.2 (0.8-6.2) | 1.9916567 | 0.2699699 |
| (MDC-STM-003-0.6) | Control | 84 | 4 | 6.8 (2.5-18.8) | 5.1726904 | 0.0000023 |
| (MDC-STM-003-1.5) | Control | 84 | 4 | 12.8 (4.7-34.9) | 6.9245385 | 0.0000000 |
| (MDC-STM-001-0.6) | Control | 98 | 4 | 38.4 (8.7-169.4) | 6.7085831 | 0.0000000 |
| (MDC-STM-002-0.6) | Control | 98 | 4 | 2.7 (0.5-13.6) | 1.6602615 | 0.4587964 |
| (MDC-STM-003-0.6) | Control | 98 | 4 | 21.2 (4.7-94.6) | 5.5628717 | 0.0000003 |
| (MDC-STM-003-1.5) | Control | 98 | 4 | 36.7 (8.3-162.4) | 6.6104282 | 0.0000000 |
| (MDC-STM-001-0.6) | Control | 112 | 4 | 25.7 (6.4-103.6) | 6.3494193 | 0.0000000 |
| (MDC-STM-002-0.6) | Control | 112 | 4 | 5.6 (1.3-23.6) | 3.2765643 | 0.0092896 |
| (MDC-STM-003-0.6) | Control | 112 | 4 | 23.5 (5.8-95.2) | 6.1673392 | 0.0000000 |
| (MDC-STM-003-1.5) | Control | 112 | 4 | 26.8 (6.6-108) | 6.4270674 | 0.0000000 |
| (MDC-STM-001-0.6) | Control | 126 | 4 | 160.9 (9.3-2793.8) | 4.8561169 | 0.0000118 |
| (MDC-STM-002-0.6) | Control | 126 | 4 | 29.5 (1.6-528.9) | 3.1984047 | 0.0120573 |
| (MDC-STM-003-0.6) | Control | 126 | 4 | 156.2 (9-2712.6) | 4.8271523 | 0.0000137 |
| (MDC-STM-003-1.5) | Control | 126 | 4 | 215.2 (12.4-3727.6) | 5.1371852 | 0.0000028 |
| (MDC-STM-001-0.6) | Control | BI | 10 | 0.6 (0.2-2.3) | -0.9559426 | 0.8747113 |
| (MDC-STM-002-0.6) | Control | BI | 10 | 0.5 (0.1-1.8) | -1.5706514 | 0.5163317 |
| (MDC-STM-003-0.6) | Control | BI | 10 | 0.5 (0.2-1.9) | -1.3559929 | 0.6559403 |
| (MDC-STM-003-1.5) | Control | BI | 10 | 0.4 (0.1-1.5) | -1.9615231 | 0.2850212 |
| (MDC-STM-001-0.6) | Control | 2 | 10 | 22.3 (8.1-61.5) | 8.3298780 | 0.0000000 |
| (MDC-STM-002-0.6) | Control | 2 | 10 | 44.4 (16.1-122.5) | 10.2014974 | 0.0000000 |
| (MDC-STM-003-0.6) | Control | 2 | 10 | 44.1 (16-121.6) | 10.1883342 | 0.0000000 |
| (MDC-STM-003-1.5) | Control | 2 | 10 | 40.8 (14.8-112.8) | 9.9635258 | 0.0000000 |
| (MDC-STM-001-0.6) | Control | 17 | 10 | 22.1 (8.7-56.1) | 9.0876376 | 0.0000000 |
| (MDC-STM-002-0.6) | Control | 17 | 10 | 19.9 (7.9-50.5) | 8.7831843 | 0.0000000 |
| (MDC-STM-003-0.6) | Control | 17 | 10 | 19.5 (7.7-49.5) | 8.7173277 | 0.0000000 |
| (MDC-STM-003-1.5) | Control | 17 | 10 | 22.9 (9-57.9) | 9.1788002 | 0.0000000 |
| (MDC-STM-001-0.6) | Control | 21 | 10 | 19.4 (7.7-48.6) | 8.8005338 | 0.0000000 |
| (MDC-STM-002-0.6) | Control | 21 | 10 | 16.7 (6.7-42) | 8.3551000 | 0.0000000 |
| (MDC-STM-003-0.6) | Control | 21 | 10 | 20.7 (8.3-52) | 8.9892405 | 0.0000000 |
| (MDC-STM-003-1.5) | Control | 21 | 10 | 21.3 (8.5-53.5) | 9.0828836 | 0.0000000 |
| (MDC-STM-001-0.6) | Control | 42 | 10 | 12.1 (4.6-31.9) | 7.0314926 | 0.0000000 |
| (MDC-STM-002-0.6) | Control | 42 | 10 | 7 (2.6-18.4) | 5.4504723 | 0.0000005 |
| (MDC-STM-003-0.6) | Control | 42 | 10 | 8.9 (3.4-23.5) | 6.1803507 | 0.0000000 |
| (MDC-STM-003-1.5) | Control | 42 | 10 | 12.3 (4.7-32.5) | 7.0804487 | 0.0000000 |
| (MDC-STM-001-0.6) | Control | 56 | 10 | 14.4 (5.6-37.2) | 7.6531475 | 0.0000000 |
| (MDC-STM-002-0.6) | Control | 56 | 10 | 7.2 (2.8-18.7) | 5.6511352 | 0.0000002 |
| (MDC-STM-003-0.6) | Control | 56 | 10 | 14.5 (5.6-37.7) | 7.6599087 | 0.0000000 |
| (MDC-STM-003-1.5) | Control | 56 | 10 | 21.2 (8.2-54.9) | 8.7464041 | 0.0000000 |
| (MDC-STM-001-0.6) | Control | 70 | 10 | 12.3 (4.8-31.6) | 7.2559840 | 0.0000000 |
| (MDC-STM-002-0.6) | Control | 70 | 10 | 3.2 (1.2-8.5) | 3.2928640 | 0.0087904 |
| (MDC-STM-003-0.6) | Control | 70 | 10 | 9 (3.5-23.2) | 6.3074379 | 0.0000000 |
| (MDC-STM-003-1.5) | Control | 70 | 10 | 15.7 (6.1-40.3) | 7.9399520 | 0.0000000 |
| (MDC-STM-001-0.6) | Control | 84 | 10 | 8 (3.2-20.3) | 6.1630229 | 0.0000000 |
| (MDC-STM-002-0.6) | Control | 84 | 10 | 2.4 (1-6.3) | 2.5834060 | 0.0734027 |
| (MDC-STM-003-0.6) | Control | 84 | 10 | 5.7 (2.2-14.4) | 5.0764744 | 0.0000038 |
| (MDC-STM-003-1.5) | Control | 84 | 10 | 10.1 (4-25.4) | 6.8093812 | 0.0000000 |
| (MDC-STM-001-0.6) | Control | 98 | 10 | 10.1 (3.9-26.2) | 6.5853039 | 0.0000000 |
| (MDC-STM-002-0.6) | Control | 98 | 10 | 2.1 (0.8-5.5) | 1.9907963 | 0.2703929 |
| (MDC-STM-003-0.6) | Control | 98 | 10 | 5.7 (2.2-15.1) | 4.9284663 | 0.0000082 |
| (MDC-STM-003-1.5) | Control | 98 | 10 | 9.6 (3.7-25) | 6.4097776 | 0.0000000 |
| (MDC-STM-001-0.6) | Control | 112 | 10 | 10.9 (4-29.9) | 6.4398595 | 0.0000000 |
| (MDC-STM-002-0.6) | Control | 112 | 10 | 3.3 (1.2-9.3) | 3.1723213 | 0.0131335 |
| (MDC-STM-003-0.6) | Control | 112 | 10 | 10.7 (3.9-29.5) | 6.3876542 | 0.0000000 |
| (MDC-STM-003-1.5) | Control | 112 | 10 | 11.5 (4.2-31.6) | 6.5809723 | 0.0000000 |
| (MDC-STM-001-0.6) | Control | 126 | 10 | 34.9 (9.3-131.3) | 7.3172285 | 0.0000000 |
| (MDC-STM-002-0.6) | Control | 126 | 10 | 10.5 (2.7-40.3) | 4.7708941 | 0.0000181 |
| (MDC-STM-003-0.6) | Control | 126 | 10 | 33.2 (8.8-125.1) | 7.2074558 | 0.0000000 |
| (MDC-STM-003-1.5) | Control | 126 | 10 | 45.5 (12.1-170.7) | 7.8755092 | 0.0000000 |
| (MDC-STM-001-0.6) | Control | BI | 30 | 1.1 (0.5-2.3) | 0.3311432 | 0.9974111 |
| (MDC-STM-002-0.6) | Control | BI | 30 | 1 (0.5-2.1) | -0.0125675 | 1.0000000 |
| (MDC-STM-003-0.6) | Control | BI | 30 | 0.8 (0.4-1.8) | -0.6516467 | 0.9663867 |
| (MDC-STM-003-1.5) | Control | BI | 30 | 0.9 (0.4-1.9) | -0.4079445 | 0.9941915 |
| (MDC-STM-001-0.6) | Control | 2 | 30 | 7.4 (3.5-15.7) | 7.2400202 | 0.0000000 |
| (MDC-STM-002-0.6) | Control | 2 | 30 | 18.3 (8.6-38.9) | 10.5353421 | 0.0000000 |
| (MDC-STM-003-0.6) | Control | 2 | 30 | 18.4 (8.7-39.1) | 10.5658195 | 0.0000000 |
| (MDC-STM-003-1.5) | Control | 2 | 30 | 12.6 (5.9-26.8) | 9.1477425 | 0.0000000 |
| (MDC-STM-001-0.6) | Control | 17 | 30 | 10.6 (5.1-22.3) | 8.7066586 | 0.0000000 |
| (MDC-STM-002-0.6) | Control | 17 | 30 | 9.4 (4.5-19.7) | 8.2714953 | 0.0000000 |
| (MDC-STM-003-0.6) | Control | 17 | 30 | 9.4 (4.5-19.6) | 8.2404428 | 0.0000000 |
| (MDC-STM-003-1.5) | Control | 17 | 30 | 11.8 (5.6-24.7) | 9.0870759 | 0.0000000 |
| (MDC-STM-001-0.6) | Control | 21 | 30 | 10.1 (4.8-21.2) | 8.5489587 | 0.0000000 |
| (MDC-STM-002-0.6) | Control | 21 | 30 | 7.4 (3.5-15.4) | 7.3709439 | 0.0000000 |
| (MDC-STM-003-0.6) | Control | 21 | 30 | 10.7 (5.1-22.4) | 8.7404073 | 0.0000000 |
| (MDC-STM-003-1.5) | Control | 21 | 30 | 11.6 (5.6-24.3) | 9.0550481 | 0.0000000 |
| (MDC-STM-001-0.6) | Control | 42 | 30 | 5.9 (2.7-12.7) | 6.3153947 | 0.0000000 |
| (MDC-STM-002-0.6) | Control | 42 | 30 | 3.8 (1.8-8.2) | 4.7956175 | 0.0000160 |
| (MDC-STM-003-0.6) | Control | 42 | 30 | 4.7 (2.2-10.2) | 5.5638129 | 0.0000003 |
| (MDC-STM-003-1.5) | Control | 42 | 30 | 6.8 (3.2-14.6) | 6.8245889 | 0.0000000 |
| (MDC-STM-001-0.6) | Control | 56 | 30 | 5.5 (2.6-11.7) | 6.2391368 | 0.0000000 |
| (MDC-STM-002-0.6) | Control | 56 | 30 | 3.1 (1.5-6.6) | 4.1792820 | 0.0002827 |
| (MDC-STM-003-0.6) | Control | 56 | 30 | 6.8 (3.2-14.3) | 6.9961241 | 0.0000000 |
| (MDC-STM-003-1.5) | Control | 56 | 30 | 7.1 (3.4-15.1) | 7.1113294 | 0.0000000 |
| (MDC-STM-001-0.6) | Control | 70 | 30 | 3.5 (1.7-7.2) | 4.6389669 | 0.0000345 |
| (MDC-STM-002-0.6) | Control | 70 | 30 | 1.4 (0.7-2.9) | 1.2618253 | 0.7146990 |
| (MDC-STM-003-0.6) | Control | 70 | 30 | 2.7 (1.3-5.7) | 3.7237241 | 0.0018361 |
| (MDC-STM-003-1.5) | Control | 70 | 30 | 4.8 (2.3-9.9) | 5.8158608 | 0.0000001 |
| (MDC-STM-001-0.6) | Control | 84 | 30 | 3.6 (1.7-7.6) | 4.8051976 | 0.0000153 |
| (MDC-STM-002-0.6) | Control | 84 | 30 | 1.5 (0.7-3.2) | 1.5836401 | 0.5079043 |
| (MDC-STM-003-0.6) | Control | 84 | 30 | 2.5 (1.2-5.1) | 3.3574350 | 0.0070421 |
| (MDC-STM-003-1.5) | Control | 84 | 30 | 4.2 (2-8.7) | 5.3227185 | 0.0000010 |
| (MDC-STM-001-0.6) | Control | 98 | 30 | 3.8 (1.8-8.1) | 4.8907639 | 0.0000099 |
| (MDC-STM-002-0.6) | Control | 98 | 30 | 1.4 (0.6-2.9) | 1.1222767 | 0.7948357 |
| (MDC-STM-003-0.6) | Control | 98 | 30 | 2.8 (1.3-6) | 3.7662399 | 0.0015562 |
| (MDC-STM-003-1.5) | Control | 98 | 30 | 4.4 (2.1-9.3) | 5.3605195 | 0.0000008 |
| (MDC-STM-001-0.6) | Control | 112 | 30 | 3.7 (1.8-7.8) | 4.7881568 | 0.0000166 |
| (MDC-STM-002-0.6) | Control | 112 | 30 | 1.5 (0.7-3.2) | 1.4979596 | 0.5637848 |
| (MDC-STM-003-0.6) | Control | 112 | 30 | 3.7 (1.8-7.9) | 4.8328687 | 0.0000133 |
| (MDC-STM-003-1.5) | Control | 112 | 30 | 4.4 (2.1-9.3) | 5.4279176 | 0.0000006 |
| (MDC-STM-001-0.6) | Control | 126 | 30 | 3.9 (1.9-8.3) | 5.0130665 | 0.0000053 |
| (MDC-STM-002-0.6) | Control | 126 | 30 | 1.7 (0.8-3.6) | 1.8970277 | 0.3188277 |
| (MDC-STM-003-0.6) | Control | 126 | 30 | 4.1 (2-8.7) | 5.1720047 | 0.0000023 |
| (MDC-STM-003-1.5) | Control | 126 | 30 | 4.6 (2.2-9.7) | 5.5941222 | 0.0000002 |

B.

| **Formulation** | **Treatment arm** | **DAI** | **Follow-up**  **(days)** | **HR CI95%** | **z.ratio** | **p.value** |
| --- | --- | --- | --- | --- | --- | --- |
| mdc-STM-001(0.6) | Control | BI | 4 | 0.4 (0.1-2.3) | -1.4525092 | 0.5935112 |
| mdc-STM-002(0.6) | Control | BI | 4 | 0.3 (0-1.9) | -1.7950230 | 0.3763965 |
| mdc-STM-003(0.6) | Control | BI | 4 | 0.6 (0.1-2.6) | -0.9988111 | 0.8560128 |
| mdc-STM-003(1.5) | Control | BI | 4 | 0.5 (0.1-2.7) | -1.0328193 | 0.8402120 |
| mdc-STM-001(0.6) | Control | 2 | 4 | 75.1 (22.9-246) | 9.9245770 | 0.0000000 |
| mdc-STM-002(0.6) | Control | 2 | 4 | 72.9 (22.3-239) | 9.8587828 | 0.0000000 |
| mdc-STM-003(0.6) | Control | 2 | 4 | 101.1 (30.9-331.1) | 10.6117691 | 0.0000000 |
| mdc-STM-003(1.5) | Control | 2 | 4 | 91 (27.8-298) | 10.3768245 | 0.0000000 |
| mdc-STM-001(0.6) | Control | 7 | 4 | 134.2 (28.1-640.2) | 8.5530652 | 0.0000000 |
| mdc-STM-002(0.6) | Control | 7 | 4 | 169.1 (35.5-806.5) | 8.9586219 | 0.0000000 |
| mdc-STM-003(0.6) | Control | 7 | 4 | 178.3 (37.4-850.7) | 9.0478567 | 0.0000000 |
| mdc-STM-003(1.5) | Control | 7 | 4 | 255.2 (53.5-1217) | 9.6791985 | 0.0000000 |
| mdc-STM-001(0.6) | Control | 17 | 4 | 53.1 (15.5-181.7) | 8.8085244 | 0.0000000 |
| mdc-STM-002(0.6) | Control | 17 | 4 | 35.5 (10.4-121.7) | 7.9027325 | 0.0000000 |
| mdc-STM-003(0.6) | Control | 17 | 4 | 50.7 (14.8-173.6) | 8.7020934 | 0.0000000 |
| mdc-STM-003(1.5) | Control | 17 | 4 | 69.4 (20.3-237.1) | 9.4072719 | 0.0000000 |
| mdc-STM-001(0.6) | Control | 21 | 4 | 62.4 (17.3-225.1) | 8.7889633 | 0.0000000 |
| mdc-STM-002(0.6) | Control | 21 | 4 | 41.4 (11.4-149.7) | 7.8924001 | 0.0000000 |
| mdc-STM-003(0.6) | Control | 21 | 4 | 45.2 (12.5-163.7) | 8.0827196 | 0.0000000 |
| mdc-STM-003(1.5) | Control | 21 | 4 | 73.1 (20.3-263.7) | 9.1269221 | 0.0000000 |
| mdc-STM-001(0.6) | Control | 28 | 4 | 97 (20.3-463.2) | 7.9817790 | 0.0000000 |
| mdc-STM-002(0.6) | Control | 28 | 4 | 50.1 (10.5-240.3) | 6.8120194 | 0.0000000 |
| mdc-STM-003(0.6) | Control | 28 | 4 | 51.5 (10.7-246.8) | 6.8614600 | 0.0000000 |
| mdc-STM-003(1.5) | Control | 28 | 4 | 77 (16.1-368) | 7.5763840 | 0.0000000 |
| mdc-STM-001(0.6) | Control | 42 | 4 | 25.3 (8.2-77.9) | 7.8344226 | 0.0000000 |
| mdc-STM-002(0.6) | Control | 42 | 4 | 8.2 (2.6-25.8) | 4.9966923 | 0.0000058 |
| mdc-STM-003(0.6) | Control | 42 | 4 | 16.7 (5.4-51.8) | 6.7820428 | 0.0000000 |
| mdc-STM-003(1.5) | Control | 42 | 4 | 28.4 (9.2-87.5) | 8.1068411 | 0.0000000 |
| mdc-STM-001(0.6) | Control | 56 | 4 | 78.8 (13.6-454.9) | 6.7925446 | 0.0000000 |
| mdc-STM-002(0.6) | Control | 56 | 4 | 20.4 (3.4-121) | 4.6202949 | 0.0000377 |
| mdc-STM-003(0.6) | Control | 56 | 4 | 55.9 (9.6-324.8) | 6.2355142 | 0.0000000 |
| mdc-STM-003(1.5) | Control | 56 | 4 | 115.4 (20-665.7) | 7.3936684 | 0.0000000 |
| mdc-STM-001(0.6) | Control | 70 | 4 | 14.3 (4.1-49.8) | 5.8249608 | 0.0000001 |
| mdc-STM-002(0.6) | Control | 70 | 4 | 4.2 (1.1-15.4) | 3.0047931 | 0.0223285 |
| mdc-STM-003(0.6) | Control | 70 | 4 | 14.7 (4.2-51.1) | 5.8808958 | 0.0000000 |
| mdc-STM-003(1.5) | Control | 70 | 4 | 26.8 (7.8-92.5) | 7.2458816 | 0.0000000 |
| mdc-STM-001(0.6) | Control | 84 | 4 | 8.8 (2.6-29.8) | 4.8796195 | 0.0000105 |
| mdc-STM-002(0.6) | Control | 84 | 4 | 1.4 (0.4-5.7) | 0.7288331 | 0.9498655 |
| mdc-STM-003(0.6) | Control | 84 | 4 | 13.7 (4.1-45.9) | 5.9298691 | 0.0000000 |
| mdc-STM-003(1.5) | Control | 84 | 4 | 16.9 (5.1-56.1) | 6.4215395 | 0.0000000 |
| mdc-STM-001(0.6) | Control | 91 | 4 | 43.8 (5.4-353.9) | 4.9315684 | 0.0000081 |
| mdc-STM-002(0.6) | Control | 91 | 4 | 8.3 (0.9-73.3) | 2.6608551 | 0.0599267 |
| mdc-STM-003(0.6) | Control | 91 | 4 | 52.4 (6.5-422.1) | 5.1774268 | 0.0000022 |
| mdc-STM-003(1.5) | Control | 91 | 4 | 68.5 (8.5-550.5) | 5.5334036 | 0.0000003 |
| mdc-STM-001(0.6) | Control | 98 | 4 | 8.7 (2.3-32.5) | 4.4604133 | 0.0000801 |
| mdc-STM-002(0.6) | Control | 98 | 4 | 2.1 (0.5-8.9) | 1.3968548 | 0.6296925 |
| mdc-STM-003(0.6) | Control | 98 | 4 | 8.5 (2.3-31.8) | 4.4446592 | 0.0000861 |
| mdc-STM-003(1.5) | Control | 98 | 4 | 14.8 (4-54.2) | 5.6493493 | 0.0000002 |
| mdc-STM-001(0.6) | Control | 105 | 4 | 7.1 (2.2-23.3) | 4.5143215 | 0.0000623 |
| mdc-STM-002(0.6) | Control | 105 | 4 | 1.3 (0.3-4.9) | 0.4836030 | 0.9888789 |
| mdc-STM-003(0.6) | Control | 105 | 4 | 6.8 (2.1-22.2) | 4.4256915 | 0.0000940 |
| mdc-STM-003(1.5) | Control | 105 | 4 | 14.8 (4.6-47.2) | 6.3098761 | 0.0000000 |
| mdc-STM-001(0.6) | Control | 112 | 4 | 5.6 (1.6-20.3) | 3.6722060 | 0.0022378 |
| mdc-STM-002(0.6) | Control | 112 | 4 | 2.2 (0.6-8.8) | 1.5855045 | 0.5066966 |
| mdc-STM-003(0.6) | Control | 112 | 4 | 7.1 (2-25.1) | 4.2230989 | 0.0002335 |
| mdc-STM-003(1.5) | Control | 112 | 4 | 9.4 (2.7-33) | 4.8654735 | 0.0000113 |
| mdc-STM-001(0.6) | Control | 119 | 4 | 15.5 (3.2-76.1) | 4.6954432 | 0.0000262 |
| mdc-STM-002(0.6) | Control | 119 | 4 | 3.4 (0.6-18.7) | 1.9371514 | 0.2975453 |
| mdc-STM-003(0.6) | Control | 119 | 4 | 18.3 (3.8-89.3) | 5.0046730 | 0.0000055 |
| mdc-STM-003(1.5) | Control | 119 | 4 | 24.9 (5.1-120.6) | 5.5529337 | 0.0000003 |
| mdc-STM-001(0.6) | Control | 126 | 4 | 42.1 (5.2-340.3) | 4.8779078 | 0.0000106 |
| mdc-STM-002(0.6) | Control | 126 | 4 | 5.5 (0.6-51.2) | 2.1031035 | 0.2186133 |
| mdc-STM-003(0.6) | Control | 126 | 4 | 36.3 (4.5-293.7) | 4.6872015 | 0.0000273 |
| mdc-STM-003(1.5) | Control | 126 | 4 | 56.5 (7-454.2) | 5.2765308 | 0.0000013 |
| mdc-STM-001(0.6) | Control | BI | 10 | 0.5 (0.2-1.4) | -1.8412832 | 0.3497023 |
| mdc-STM-002(0.6) | Control | BI | 10 | 0.6 (0.2-1.6) | -1.4389514 | 0.6023572 |
| mdc-STM-003(0.6) | Control | BI | 10 | 0.7 (0.3-1.8) | -1.1321622 | 0.7895059 |
| mdc-STM-003(1.5) | Control | BI | 10 | 0.7 (0.3-1.9) | -0.9502176 | 0.8771030 |
| mdc-STM-001(0.6) | Control | 2 | 10 | 23.7 (10.5-53.7) | 10.5671244 | 0.0000000 |
| mdc-STM-002(0.6) | Control | 2 | 10 | 24.5 (10.8-55.4) | 10.6674750 | 0.0000000 |
| mdc-STM-003(0.6) | Control | 2 | 10 | 34.7 (15.3-78.6) | 11.8402697 | 0.0000000 |
| mdc-STM-003(1.5) | Control | 2 | 10 | 31.4 (13.9-71.1) | 11.5091218 | 0.0000000 |
| mdc-STM-001(0.6) | Control | 7 | 10 | 21.8 (9.5-49.9) | 10.1539733 | 0.0000000 |
| mdc-STM-002(0.6) | Control | 7 | 10 | 28.2 (12.3-64.4) | 10.9963707 | 0.0000000 |
| mdc-STM-003(0.6) | Control | 7 | 10 | 29.5 (12.9-67.5) | 11.1369242 | 0.0000000 |
| mdc-STM-003(1.5) | Control | 7 | 10 | 43.3 (18.9-99) | 12.4087000 | 0.0000000 |
| mdc-STM-001(0.6) | Control | 17 | 10 | 12.4 (5.6-27.6) | 8.6218624 | 0.0000000 |
| mdc-STM-002(0.6) | Control | 17 | 10 | 8.3 (3.7-18.5) | 7.2272830 | 0.0000000 |
| mdc-STM-003(0.6) | Control | 17 | 10 | 12.4 (5.6-27.5) | 8.6164337 | 0.0000000 |
| mdc-STM-003(1.5) | Control | 17 | 10 | 17.5 (7.9-38.8) | 9.7930817 | 0.0000000 |
| mdc-STM-001(0.6) | Control | 21 | 10 | 11.1 (5.1-24.4) | 8.3818904 | 0.0000000 |
| mdc-STM-002(0.6) | Control | 21 | 10 | 8 (3.6-17.6) | 7.2161276 | 0.0000000 |
| mdc-STM-003(0.6) | Control | 21 | 10 | 9.1 (4.1-19.9) | 7.6585295 | 0.0000000 |
| mdc-STM-003(1.5) | Control | 21 | 10 | 12.9 (5.9-28.4) | 8.8957932 | 0.0000000 |
| mdc-STM-001(0.6) | Control | 28 | 10 | 19.3 (8.1-45.7) | 9.3607437 | 0.0000000 |
| mdc-STM-002(0.6) | Control | 28 | 10 | 10.5 (4.4-25.1) | 7.4189722 | 0.0000000 |
| mdc-STM-003(0.6) | Control | 28 | 10 | 10.6 (4.5-25.3) | 7.4419849 | 0.0000000 |
| mdc-STM-003(1.5) | Control | 28 | 10 | 15.9 (6.7-37.6) | 8.7267934 | 0.0000000 |
| mdc-STM-001(0.6) | Control | 42 | 10 | 10.4 (4.5-24.1) | 7.6317937 | 0.0000000 |
| mdc-STM-002(0.6) | Control | 42 | 10 | 3.8 (1.6-8.9) | 4.2488554 | 0.0002084 |
| mdc-STM-003(0.6) | Control | 42 | 10 | 7.2 (3.1-16.8) | 6.3764610 | 0.0000000 |
| mdc-STM-003(1.5) | Control | 42 | 10 | 12.9 (5.6-29.7) | 8.3139612 | 0.0000000 |
| mdc-STM-001(0.6) | Control | 56 | 10 | 28.4 (10.1-80.2) | 8.8128648 | 0.0000000 |
| mdc-STM-002(0.6) | Control | 56 | 10 | 8.2 (2.8-23.6) | 5.4098301 | 0.0000006 |
| mdc-STM-003(0.6) | Control | 56 | 10 | 19.1 (6.7-54.4) | 7.7040423 | 0.0000000 |
| mdc-STM-003(1.5) | Control | 56 | 10 | 39.7 (14.1-111.8) | 9.6938839 | 0.0000000 |
| mdc-STM-001(0.6) | Control | 70 | 10 | 12.9 (4.6-36) | 6.8149255 | 0.0000000 |
| mdc-STM-002(0.6) | Control | 70 | 10 | 4.4 (1.5-12.6) | 3.8067849 | 0.0013268 |
| mdc-STM-003(0.6) | Control | 70 | 10 | 12.5 (4.5-34.9) | 6.7040907 | 0.0000000 |
| mdc-STM-003(1.5) | Control | 70 | 10 | 21.4 (7.7-59.5) | 8.1851871 | 0.0000000 |
| mdc-STM-001(0.6) | Control | 84 | 10 | 2.9 (1.3-6.7) | 3.5562395 | 0.0034569 |
| mdc-STM-002(0.6) | Control | 84 | 10 | 1.2 (0.5-2.9) | 0.6589596 | 0.9650042 |
| mdc-STM-003(0.6) | Control | 84 | 10 | 4.2 (1.9-9.6) | 4.8024106 | 0.0000155 |
| mdc-STM-003(1.5) | Control | 84 | 10 | 5.3 (2.4-12.1) | 5.6207999 | 0.0000002 |
| mdc-STM-001(0.6) | Control | 91 | 10 | 5.7 (2.3-14) | 5.3194529 | 0.0000010 |
| mdc-STM-002(0.6) | Control | 91 | 10 | 2.1 (0.8-5.3) | 2.1260924 | 0.2088806 |
| mdc-STM-003(0.6) | Control | 91 | 10 | 6.6 (2.7-16.2) | 5.7875928 | 0.0000001 |
| mdc-STM-003(1.5) | Control | 91 | 10 | 7.7 (3.2-18.8) | 6.2545248 | 0.0000000 |
| mdc-STM-001(0.6) | Control | 98 | 10 | 5.4 (2.1-13.8) | 4.9224411 | 0.0000085 |
| mdc-STM-002(0.6) | Control | 98 | 10 | 2.4 (0.9-6.3) | 2.4565738 | 0.1007138 |
| mdc-STM-003(0.6) | Control | 98 | 10 | 5.4 (2.1-13.7) | 4.9189045 | 0.0000086 |
| mdc-STM-003(1.5) | Control | 98 | 10 | 8.1 (3.2-20.3) | 6.1510916 | 0.0000000 |
| mdc-STM-001(0.6) | Control | 105 | 10 | 3.8 (1.6-9.1) | 4.1562642 | 0.0003123 |
| mdc-STM-002(0.6) | Control | 105 | 10 | 1.8 (0.7-4.3) | 1.7014145 | 0.4329995 |
| mdc-STM-003(0.6) | Control | 105 | 10 | 4.2 (1.8-10.1) | 4.5216255 | 0.0000602 |
| mdc-STM-003(1.5) | Control | 105 | 10 | 9.2 (3.9-21.7) | 7.0669937 | 0.0000000 |
| mdc-STM-001(0.6) | Control | 112 | 10 | 3.6 (1.5-8.7) | 3.8574151 | 0.0010845 |
| mdc-STM-002(0.6) | Control | 112 | 10 | 2.1 (0.8-5.2) | 2.1245354 | 0.2095304 |
| mdc-STM-003(0.6) | Control | 112 | 10 | 4.2 (1.7-10.1) | 4.3546434 | 0.0001299 |
| mdc-STM-003(1.5) | Control | 112 | 10 | 4.4 (1.8-10.8) | 4.5611161 | 0.0000500 |
| mdc-STM-001(0.6) | Control | 119 | 10 | 6.2 (2.3-16.6) | 5.0086104 | 0.0000054 |
| mdc-STM-002(0.6) | Control | 119 | 10 | 2.2 (0.8-6.2) | 2.0603323 | 0.2375089 |
| mdc-STM-003(0.6) | Control | 119 | 10 | 6.2 (2.3-16.7) | 5.0217955 | 0.0000051 |
| mdc-STM-003(1.5) | Control | 119 | 10 | 9.9 (3.7-26.5) | 6.4010372 | 0.0000000 |
| mdc-STM-001(0.6) | Control | 126 | 10 | 8.6 (3.3-22) | 6.2106643 | 0.0000000 |
| mdc-STM-002(0.6) | Control | 126 | 10 | 2.2 (0.8-6.1) | 2.1991847 | 0.1799034 |
| mdc-STM-003(0.6) | Control | 126 | 10 | 6.9 (2.7-17.7) | 5.5486393 | 0.0000003 |
| mdc-STM-003(1.5) | Control | 126 | 10 | 9.4 (3.6-24) | 6.4624035 | 0.0000000 |
| mdc-STM-001(0.6) | Control | BI | 30 | 0.9 (0.5-1.7) | -0.4413259 | 0.9921471 |
| mdc-STM-002(0.6) | Control | BI | 30 | 0.8 (0.4-1.6) | -0.7137346 | 0.9534396 |
| mdc-STM-003(0.6) | Control | BI | 30 | 0.7 (0.4-1.4) | -1.3516314 | 0.6587202 |
| mdc-STM-003(1.5) | Control | BI | 30 | 0.7 (0.4-1.4) | -1.3346408 | 0.6695032 |
| mdc-STM-001(0.6) | Control | 2 | 30 | 9.5 (5-18.1) | 9.5963295 | 0.0000000 |
| mdc-STM-002(0.6) | Control | 2 | 30 | 12.6 (6.7-24) | 10.7821368 | 0.0000000 |
| mdc-STM-003(0.6) | Control | 2 | 30 | 15.2 (8-28.9) | 11.5763541 | 0.0000000 |
| mdc-STM-003(1.5) | Control | 2 | 30 | 15.5 (8.2-29.5) | 11.6599028 | 0.0000000 |
| mdc-STM-001(0.6) | Control | 7 | 30 | 9.9 (5.2-18.9) | 9.7065456 | 0.0000000 |
| mdc-STM-002(0.6) | Control | 7 | 30 | 11.7 (6.1-22.2) | 10.3959345 | 0.0000000 |
| mdc-STM-003(0.6) | Control | 7 | 30 | 13.1 (6.9-24.9) | 10.8754247 | 0.0000000 |
| mdc-STM-003(1.5) | Control | 7 | 30 | 20.5 (10.7-39) | 12.7590423 | 0.0000000 |
| mdc-STM-001(0.6) | Control | 17 | 30 | 7.1 (3.8-13.5) | 8.3577625 | 0.0000000 |
| mdc-STM-002(0.6) | Control | 17 | 30 | 4.3 (2.3-8.2) | 6.2506357 | 0.0000000 |
| mdc-STM-003(0.6) | Control | 17 | 30 | 6.5 (3.4-12.4) | 7.9786739 | 0.0000000 |
| mdc-STM-003(1.5) | Control | 17 | 30 | 10.6 (5.6-20.2) | 10.0472269 | 0.0000000 |
| mdc-STM-001(0.6) | Control | 21 | 30 | 7.6 (4-14.4) | 8.6089502 | 0.0000000 |
| mdc-STM-002(0.6) | Control | 21 | 30 | 5 (2.6-9.5) | 6.8561647 | 0.0000000 |
| mdc-STM-003(0.6) | Control | 21 | 30 | 5.2 (2.7-9.9) | 6.9905957 | 0.0000000 |
| mdc-STM-003(1.5) | Control | 21 | 30 | 8.9 (4.7-16.9) | 9.2674039 | 0.0000000 |
| mdc-STM-001(0.6) | Control | 28 | 30 | 6.8 (3.6-13) | 8.1629184 | 0.0000000 |
| mdc-STM-002(0.6) | Control | 28 | 30 | 3.5 (1.8-6.7) | 5.3356150 | 0.0000009 |
| mdc-STM-003(0.6) | Control | 28 | 30 | 3.5 (1.9-6.7) | 5.3392816 | 0.0000009 |
| mdc-STM-003(1.5) | Control | 28 | 30 | 5.6 (2.9-10.6) | 7.2901991 | 0.0000000 |
| mdc-STM-001(0.6) | Control | 42 | 30 | 5.5 (2.9-10.7) | 7.0734936 | 0.0000000 |
| mdc-STM-002(0.6) | Control | 42 | 30 | 2.2 (1.1-4.2) | 3.1845824 | 0.0126174 |
| mdc-STM-003(0.6) | Control | 42 | 30 | 4.4 (2.3-8.4) | 6.0755004 | 0.0000000 |
| mdc-STM-003(1.5) | Control | 42 | 30 | 6.3 (3.2-12.1) | 7.5618084 | 0.0000000 |
| mdc-STM-001(0.6) | Control | 56 | 30 | 4.8 (2.5-9.1) | 6.6095855 | 0.0000000 |
| mdc-STM-002(0.6) | Control | 56 | 30 | 1.8 (0.9-3.4) | 2.4143863 | 0.1113955 |
| mdc-STM-003(0.6) | Control | 56 | 30 | 3.4 (1.8-6.5) | 5.1304999 | 0.0000029 |
| mdc-STM-003(1.5) | Control | 56 | 30 | 7 (3.7-13.4) | 8.2612435 | 0.0000000 |
| mdc-STM-001(0.6) | Control | 70 | 30 | 2.8 (1.5-5.3) | 4.3426810 | 0.0001371 |
| mdc-STM-002(0.6) | Control | 70 | 30 | 1.5 (0.8-2.8) | 1.5709813 | 0.5161174 |
| mdc-STM-003(0.6) | Control | 70 | 30 | 2.5 (1.3-4.7) | 3.8103196 | 0.0013084 |
| mdc-STM-003(1.5) | Control | 70 | 30 | 4 (2.1-7.6) | 5.8398598 | 0.0000001 |
| mdc-STM-001(0.6) | Control | 84 | 30 | 1.6 (0.9-3.1) | 2.1039597 | 0.2182455 |
| mdc-STM-002(0.6) | Control | 84 | 30 | 1.1 (0.6-2.2) | 0.5889268 | 0.9767710 |
| mdc-STM-003(0.6) | Control | 84 | 30 | 2.4 (1.3-4.6) | 3.8137967 | 0.0012905 |
| mdc-STM-003(1.5) | Control | 84 | 30 | 2.7 (1.4-5.2) | 4.2572940 | 0.0002008 |
| mdc-STM-001(0.6) | Control | 91 | 30 | 1.9 (1-3.7) | 2.7988980 | 0.0410041 |
| mdc-STM-002(0.6) | Control | 91 | 30 | 1.2 (0.6-2.2) | 0.6465748 | 0.9673238 |
| mdc-STM-003(0.6) | Control | 91 | 30 | 2.3 (1.2-4.4) | 3.5590342 | 0.0034214 |
| mdc-STM-003(1.5) | Control | 91 | 30 | 2.5 (1.3-4.9) | 3.9686477 | 0.0006898 |
| mdc-STM-001(0.6) | Control | 98 | 30 | 2 (1.1-3.9) | 3.0181363 | 0.0214296 |
| mdc-STM-002(0.6) | Control | 98 | 30 | 1.2 (0.6-2.3) | 0.8332180 | 0.9203662 |
| mdc-STM-003(0.6) | Control | 98 | 30 | 2 (1.1-3.8) | 2.9714118 | 0.0247237 |
| mdc-STM-003(1.5) | Control | 98 | 30 | 2.5 (1.3-4.8) | 3.9617328 | 0.0007098 |
| mdc-STM-001(0.6) | Control | 105 | 30 | 1.4 (0.8-2.7) | 1.5660574 | 0.5193176 |
| mdc-STM-002(0.6) | Control | 105 | 30 | 1.1 (0.6-2.1) | 0.4346779 | 0.9925898 |
| mdc-STM-003(0.6) | Control | 105 | 30 | 1.7 (0.9-3.3) | 2.3262059 | 0.1365363 |
| mdc-STM-003(1.5) | Control | 105 | 30 | 2.9 (1.5-5.4) | 4.5183891 | 0.0000611 |
| mdc-STM-001(0.6) | Control | 112 | 30 | 1.6 (0.9-3.1) | 2.1173510 | 0.2125465 |
| mdc-STM-002(0.6) | Control | 112 | 30 | 1.3 (0.7-2.4) | 0.9586987 | 0.8735510 |
| mdc-STM-003(0.6) | Control | 112 | 30 | 1.7 (0.9-3.2) | 2.1785290 | 0.1877897 |
| mdc-STM-003(1.5) | Control | 112 | 30 | 2.1 (1.1-4) | 3.1795246 | 0.0128281 |
| mdc-STM-001(0.6) | Control | 119 | 30 | 1.9 (1-3.7) | 2.7986785 | 0.0410296 |
| mdc-STM-002(0.6) | Control | 119 | 30 | 1.3 (0.7-2.5) | 1.1645890 | 0.7716195 |
| mdc-STM-003(0.6) | Control | 119 | 30 | 1.9 (1-3.7) | 2.7812962 | 0.0430913 |
| mdc-STM-003(1.5) | Control | 119 | 30 | 2.6 (1.4-5) | 4.0242898 | 0.0005475 |
| mdc-STM-001(0.6) | Control | 126 | 30 | 2.2 (1.2-4.2) | 3.3472055 | 0.0072961 |
| mdc-STM-002(0.6) | Control | 126 | 30 | 1 (0.5-2) | 0.1792798 | 0.9997694 |
| mdc-STM-003(0.6) | Control | 126 | 30 | 1.9 (1-3.6) | 2.7780905 | 0.0434810 |
| mdc-STM-003(1.5) | Control | 126 | 30 | 2.2 (1.1-4.1) | 3.2770919 | 0.0092731 |

### Supplementary table S9. Between formulations comparisons of 4-day LC50 and L90 values for KIS mosquitoes fed on treated cattle.

| Comparison | Estimate | Std. Error | t-value | p-value |
| --- | --- | --- | --- | --- |
| 001-0.6/002-0.6:50/50 | 0.8867454 | 0.5245928 | -0.2158904 | 0.8290732 |
| 001-0.6/003-0.6:50/50 | 0.9276367 | 0.5590509 | -0.1294396 | 0.8970098 |
| 001-0.6/003-1.5:50/50 | 0.9299036 | 0.5664743 | -0.1237415 | 0.9015200 |
| 002-0.6/003-0.6:50/50 | 1.0461138 | 0.3575767 | 0.1289621 | 0.8973877 |
| 002-0.6/003-1.5:50/50 | 1.0486704 | 0.3703614 | 0.1314131 | 0.8954485 |
| 003-0.6/003-1.5:50/50 | 1.0024438 | 0.3723224 | 0.0065637 | 0.9947629 |
| 001-0.6/002-0.6:90/90 | 1.3316348 | 0.7287634 | 0.4550651 | 0.6490624 |
| 001-0.6/003-0.6:90/90 | 1.1868356 | 0.5802706 | 0.3219801 | 0.7474678 |
| 001-0.6/003-1.5:90/90 | 0.9649750 | 0.4975064 | -0.0704012 | 0.9438744 |
| 002-0.6/003-0.6:90/90 | 0.8912621 | 0.4205113 | -0.2585850 | 0.7959554 |
| 002-0.6/003-1.5:90/90 | 0.7246544 | 0.3618711 | -0.7608942 | 0.4467203 |
| 003-0.6/003-1.5:90/90 | 0.8130654 | 0.3533909 | -0.5289739 | 0.5968236 |

001 = mdc-STM-001

002 = mdc-STM-002

003 = mdc-STM-003

### Supplementary Table S10. Between formulations comparisons of 10-day LC50 and LC90 values for KIS mosquitoes fed on treated cattle.

| Comparison | Estimate | Std. Error | t-value | p-value |
| --- | --- | --- | --- | --- |
| 001-0.6/002-0.6:50/50 | 0.8503147 | 0.4901133 | -0.3054095 | 0.7600542 |
| 001-0.6/003-0.6:50/50 | 0.8484447 | 0.4406361 | -0.3439467 | 0.7308864 |
| 001-0.6/003-1.5:50/50 | 0.9622920 | 0.5826786 | -0.0647150 | 0.9484009 |
| 002-0.6/003-0.6:50/50 | 0.9978008 | 0.4234921 | -0.0051931 | 0.9958566 |
| 002-0.6/003-1.5:50/50 | 1.1316892 | 0.5956824 | 0.2210728 | 0.8250358 |
| 003-0.6/003-1.5:50/50 | 1.1341835 | 0.5253544 | 0.2554152 | 0.7984025 |
| 001-0.6/002-0.6:90/90 | 0.9526083 | 0.5708619 | -0.0830179 | 0.9338373 |
| 001-0.6/003-0.6:90/90 | 1.1594824 | 0.5445723 | 0.2928580 | 0.7696307 |
| 001-0.6/003-1.5:90/90 | 1.0292734 | 0.5106321 | 0.0573278 | 0.9542841 |
| 002-0.6/003-0.6:90/90 | 1.2171660 | 0.6993343 | 0.3105324 | 0.7561561 |
| 002-0.6/003-1.5:90/90 | 1.0804792 | 0.6443631 | 0.1248973 | 0.9006048 |
| 003-0.6/003-1.5:90/90 | 0.8877008 | 0.4136421 | -0.2714888 | 0.7860151 |

### Supplementary Table S11. Between formulations comparisons of 4-day LC50 and LC90 values for VK5 mosquitoes fed on treated cattle.

| Comparison | Estimate | Std. Error | t-value | p-value |
| --- | --- | --- | --- | --- |
| 001-0.6/002-0.6:50/50 | 0.8629191 | 0.2962409 | -0.4627344 | 0.6435547 |
| 001-0.6/003-0.6:50/50 | 0.9532895 | 0.2969513 | -0.1573003 | 0.8750082 |
| 001-0.6/003-1.5:50/50 | 0.9981696 | 0.3163292 | -0.0057865 | 0.9953830 |
| 002-0.6/003-0.6:50/50 | 1.1047263 | 0.3647694 | 0.2871029 | 0.7740335 |
| 002-0.6/003-1.5:50/50 | 1.1567359 | 0.3878491 | 0.4041157 | 0.6861277 |
| 003-0.6/003-1.5:50/50 | 1.0470792 | 0.3169059 | 0.1485588 | 0.8819018 |
| 001-0.6/002-0.6:90/90 | 0.8442851 | 0.4320073 | -0.3604451 | 0.7185143 |
| 001-0.6/003-0.6:90/90 | 0.9480422 | 0.4348192 | -0.1194928 | 0.9048849 |
| 001-0.6/003-1.5:90/90 | 1.0899361 | 0.4614506 | 0.1948987 | 0.8454723 |
| 002-0.6/003-0.6:90/90 | 1.1228935 | 0.5912852 | 0.2078414 | 0.8353528 |
| 002-0.6/003-1.5:90/90 | 1.2909575 | 0.6405087 | 0.4542600 | 0.6496417 |
| 003-0.6/003-1.5:90/90 | 1.1496704 | 0.5072949 | 0.2950363 | 0.7679661 |

### Supplementary table S12. Between formulations comparisons of 10-day LC50 and LC90 values for VK5 mosquitoes fed on treated cattle.

| Comparison | Estimate | Std. Error | t-value | p-value |
| --- | --- | --- | --- | --- |
| 001-0.6/002-0.6:50/50 | 0.9513449 | 0.4334657 | -0.1122468 | 0.9106277 |
| 001-0.6/003-0.6:50/50 | 0.9647061 | 0.4190361 | -0.0842264 | 0.9328765 |
| 001-0.6/003-1.5:50/50 | 0.9823391 | 0.4071531 | -0.0433767 | 0.9654013 |
| 002-0.6/003-0.6:50/50 | 1.0140446 | 0.4935095 | 0.0284586 | 0.9772964 |
| 002-0.6/003-1.5:50/50 | 1.0325793 | 0.4842848 | 0.0672731 | 0.9463643 |
| 003-0.6/003-1.5:50/50 | 1.0182780 | 0.4565668 | 0.0400337 | 0.9680663 |
| 001-0.6/002-0.6:90/90 | 0.9165698 | 0.5121319 | -0.1629076 | 0.8705911 |
| 001-0.6/003-0.6:90/90 | 1.0141546 | 0.4742174 | 0.0298484 | 0.9761880 |
| 001-0.6/003-1.5:90/90 | 1.0997614 | 0.5007874 | 0.1992090 | 0.8420993 |
| 002-0.6/003-0.6:90/90 | 1.1064674 | 0.6179795 | 0.1722831 | 0.8632150 |
| 002-0.6/003-1.5:90/90 | 1.1998665 | 0.6578991 | 0.3037950 | 0.7612841 |
| 003-0.6/003-1.5:90/90 | 1.0844119 | 0.4934883 | 0.1710515 | 0.8641833 |

Supplementary Table S13. Lethal concentration (LC50) for KIS and VK5 mosquitoes over 4-day follow up period (4-day LC50). Mean concentrations are given with lower and upper confidence intervals (95% CI).

| Cumulative mortality window (days) | Mortality % | KIS mosquitoes | VK5 mosquitoes | *P-value* (between colony comparison) |
| --- | --- | --- | --- | --- |
| 4 | 50 | 2.89 [2.27-3.67] | 5.26 [4.36-6.35] | 1.0e-07 |
| 4 | 90 | 9.2 [6.72-12.59] | 17.65 [12.86-24.22] | 0.0e+00 |
