# Supplementary material for: Selection of the optimal long-acting injectable formulation of ivermectin for use in humans to target malaria vectors in Western Africa: evaluation of pharmacokinetics and mosquitocidal efficacy in cattle under laboratory conditions
Source: Parasit Vectors. 2026 May 6;19:226. doi: 10.1186/s13071-026-07263-x (PMC13192019; doi:10.1186/s13071-026-07263-x)
Supplement: Supplementary file 2 — Additional file 2: Figure S1. Additional mean concentration–time profiles of ivermectin in cattle plasma. Figure S2. Kaplan-Meier plots. Figure S3. Four-day cumulative mortality of KIS and VK5. Figure S4. Heat maps illustrating the lower 95% confidence limit (LCL) of 4-day and 30-day mortality hazard ratios (HRs) for KIS and VK5 colony mosquitoes. Figure S5. Relationship between ivermectin plasma concentration and 4-day (A, B) or 10-day (C , D) cumulative mortality for each candidate formulation. Figure S6. Relationship between ivermectin plasma concentrations and 4-day cumulative mosquito mortality for KIS and VK5 colonies [file 13071_2026_7263_MOESM2_ESM.docx]

Supplementary Fig. S1: Additional mean concentration–time profiles of ivermectin in cattle plasma. Panels A and C: profiles by formulation at a dose of 0.6 mg/kg for the entire experiment (A) and during the first 7 days post-injection to visualize the initial burst (C). Panels B and D: profiles by dose for the entire experiment (B) and during the first 7 days post-injection (D)

Supplementary Fig. S2. Kaplan-Meier plots illustrating the mosquitocidal effects of the candidate long-lasting ivermectin formulations administered to calves at doses of 0.6 mg/kg (mdc-STM-001, mdc-STM-002 and mdc-STM-003) or 1.5 mg/kg (mdc-STM-003 only). The plots depict the results from each direct skin feeding assay conducted at the indicated time points (Days After Injection, DAI). Mosquito survival was monitored daily for up to 30 days post-blood meal. Pannel A: KIS colony, Pannel B: VK5 colony.

Supplementary Fig. S3: 4-day cumulative mortality of KIS and VK5 colony mosquitoes after feeding on calves treated using the candidate formulations. Treated cattle received a single LAIF injection at a dose of 0.6mg/kg for the mdc-STM-001, mdc-STM-002, mdc-STM-003 formulations, and at an additional dose of 1.5 mg/kg for the mdc-STM-003. The smooth line represents mean values estimated by the LOESS (locally estimated scatterplot smoothing) method and grey area shows CI95%. See the main text for further details.

Supplementary Fig. S4. Heat maps illustrating the lower 95% confidence limit (LCL) of 4-day and 30-day mortality hazard ratios (HRs) for KIS and VK5 colony mosquitoes fed at different DAIs on cattle treated with the formulations mdc-STM-001-0.6, mdc-STM-002-0.6, mdc-STM-003-0.6 or mdc-STM-003-1.5. HR and *P-values* for each DAI are available in Additional file 1: table S7. BI is the timepoint before injection.

Supplementary Fig. S5. Relationship between ivermectin plasma concentration and 4-days (A and B) or 10-day (C and D) cumulative mortality for each candidate formulation. A and C: KIS colony mosquitoes. B and C: VK5 colony mosquitoes

Supplementary Fig.6. Relationship between ivermectin plasma concentrations and 4-day cumulative mosquito mortality for KIS and VK5 colonies.
